# Effects of different exercise training modes on muscle strength and physical performance in older people with sarcopenia: a systematic review and meta-analysis

**DOI:** 10.1186/s12877-021-02642-8

**Published:** 2021-12-15

**Authors:** Linqian Lu, Lin Mao, Yuwei Feng, Barbara E. Ainsworth, Yu Liu, Nan Chen

**Affiliations:** 1 Key Laboratory of Exercise and Health Sciences of Ministry of Education, Shanghai University of Sport, Shanghai, 200438 China; 2 Department of Rehabilitation, Xinhua Hospital Chongming Branch, Shanghai, 202150 China; 3 Department of Rehabilitation, Xinhua Hospital Affiliated to Shanghai Jiaotong University School of Medicine, Shanghai, China; 4 College of Health Solutions, Arizona State University, Phoenix, AZ USA

**Keywords:** Sarcopenia, Resistance training, Whole body vibration training, Mixed training, Muscle strength, Physical performance

## Abstract

**Objective:**

We conducted a systematic review and meta-analysis to clarify the effects of different exercise modes (resistance training [RT], whole body vibration training [WBVT], and mixed training [MT, resistance training combined with other exercises such as balance, endurance and aerobic training]) on muscle strength (knee extension strength [KES]) and physical performance (Timed Up and Go [TUG], gait speed [GS] and the Chair Stand [CS]) in older people with sarcopenia.

**Method:**

All studies published from January 2010 to March 2021 on the effects of exercise training in older people with sarcopenia were retrieved from 6 electronic databases: Pubmed, Cochrane Library, Embase, Web of Science, the China National Knowledge Infrastructure (CNKI), and Wanfang Database. Two researchers independently extracted and evaluated studies that met inclusion and exclusion criteria. Pooled analyses for pre- and post- outcome measurements were performed using Review Manager 5.4 with standardized mean differences (SMDs) and fixed-effect models.

**Result:**

Twenty-six studies (25 randomized controlled trails [RCTs] and one non-randomized controlled trail) were included in this study with 1191 older people with sarcopenia (mean age 60.6 ± 2.3 to 89.5 ± 4.4). Compared with a control group, RT and MT significantly improved KES (RT, SMD = 1.36, 95% confidence intervals [95% CI]: 0.71 to 2.02, *p* < 0.0001, I^2^ = 72%; MT, SMD = 0.62, 95% CI: 0.29 to 0.95, *p* = 0.0002, I^2^ = 56%) and GS (RT, SMD = 2.01, 95% CI: 1.04 to 2.97, *p* < 0.0001, I^2^ = 84%; MT, SMD = 0.69, 95% CI: 0.29 to 1.09, *p* = 0.008, I^2^ = 81%). WBVT showed no changes in KES (SMD = 0.65, 95% CI: − 0.02 to 1.31, *p* = 0.06, I^2^ = 80%) or GS (SMD = 0.12, 95% CI: − 0.15 to 0.39, *p* = 0.38, I^2^ = 0%). TUG times were significantly improved with all exercise training modes (SMD = -0.66, 95% CI: − 0.94 to − 0.38, *p* < 0.00001, I^2^ = 60%). There were no changes in CS times with any of the exercise training modes (SMD = 0.11, 95% CI: − 0.36 to 0.57, *p* = 0.65, I^2^ = 87%).

**Conclusions:**

In older people with sarcopenia, KES and GS can be improved by RT and MT, but not by WBVT. All three training modes improved TUG times, but not improved CS times.

**Supplementary Information:**

The online version contains supplementary material available at 10.1186/s12877-021-02642-8.

## Background

Sarcopenia is an age-dependent geriatric syndrome characterized by skeletal muscle mass loss, muscle strength and/or declines in physical performance [1].

A meta-analysis in 2020 indicated that the prevalence of sarcopenia was 9–10% in community-dwelling individuals, 30–50% in nursing-home individuals and 23–24% in hospitalized individuals [2]. Sarcopenia is associated with several adverse outcomes, including falls and secondary fractures [3, 4], pulmonary insufficiency [5], sleep disorders [6], cognitive impairment [7], poor quality of health-life [8] and premature mortality [9], all of which bring significant medical and economic burdens. Sarcopenia increases the risk of hospitalization of older people [10, 11]. Total hospitalization costs are higher by nearly $13,000 in preoperative older people with sarcopenia compared to older people without sarcopenia [12]. In 2000, the costs for sarcopenia-related conditions in the United States was $18.5 billion, which represented 1.5% of the annual medical expenditures [13]. Therefore, the prevention and treatment for sarcopenia is important to maintain physical function and improve health outcomes for older people and to reduce medical expenditures associated with sarcopenia.

To date, there are no effective pharmacological interventions to the treatment of sarcopenia [14]. Non-pharmacological interventions are the most appropriate and effective intervention for sarcopenia [15]. As a non-pharmacological intervention, exercise has been demonstrated by randomized controlled trails [RCTs] and meta-analysis to produce significant physiological and health benefits and to prevent and/or delay the development of sarcopenia [16–18]. The American College of Sports Medicine and the World Health Organization recommends that older people maintain 150 min of moderate-intensity aerobic exercise per week or 75 min of high-intensity aerobic exercise per week, and perform resistance exercise 2–3 times per week to prevent chronic or debilitating conditions and/or treat disease [19, 20]. Previous studies have demonstrated that exercise (e.g., resistance training [RT], whole body vibration training [WBVT], mixed training [MT, such as resistance training combined with balance and aerobic training]) have a positive effect on increasing muscle mass [21], muscle strength [22] and physical performance [23]) in older people with sarcopenia.

Evidence has been provided from systematic reviews, meta-analyses and RCTs to show the efficacy of different exercise training modes on muscle mass, muscle strength and physical performance in older people with sarcopenia. Two reviews showed that RT can improve muscle strength and physical performance [24, 25] and that MT can increase physical performance [26]. Two meta-analyses also showed that RT is effective in improving muscle strength and physical performance and that WBVT has a positive effect on physical performance [27, 28]. To date, little is known about the similarities and differences of RT, WBVT, MT exercise training modes and the effects of the study designs and/or protocols on muscle strength and physical performance.

Various methodologic and design weaknesses have consistently been found in exercise studies that limit the dissemination of exercise findings among older people with sarcopenia. First, there is no consensus on sarcopenia diagnostic criteria that makes studies included in previous meta-analysis inconsistent for the diagnostic criteria of sarcopenia [29]. In addition, there are differences in measurement methods for muscle strength and physical performance measures [30, 31], and exercise protocols [32, 33] that can lead to high heterogeneity in the results. Second, few studies have investigated the efficacy of different exercise modes to improve health outcomes and fitness levels in older people with sarcopenia**.** Alternatively, studies have focused on improving health outcomes and fitness in healthy older people [34], older people with osteoarthritis [35] or osteoporosis [36] or heart failure [37]. The aim of this study is to investigate the efficacy of three exercise training modes (RT, WBVT and MT) on knee extension strength (KES) and physical performance tests (Timed Up and Go [TUG], gait speed [GS] and the Chair Stand [CS]) to provide additional evidence for the treatment and management of sarcopenia in older people.

## Methods

### Eligibility criteria

The studies were included if they met the criteria for subjects and study types as follows: (a) age > 60 years; (b) diagnosed with sarcopenia; (c) without diseases or conditions of COPD, cancer, kidney disease, hypertension, hyperlipidemia, stroke, diabetes, obesity, osteoporosis and fracture; (d) the study has at least one exercise group and one control group, and the control group must receive a no-exercise intervention or a health education course; (e) the exercise group must contain at least one type of RT, WBVT and/or MT exercise modes; and (f) each included study is the latest report since 2010.

The studies were excluded if they failed to meet the inclusion criteria and/or: (a) not full-text; (b) not in English or Chinese; (c) not a randomized controlled trial (RCT); (d) subjects were not diagnosed with sarcopenia previously in the exercise group and control group; (e) the exercise group received exercise interventions combined with nutritional supplementation; and (f) the study presented no extractable data.

### Search strategy

This systematic review and meta-analysis is registered on the International Prospective Register of Systematic Reviews site (PROSPERO) as CRD42021256110. We followed the Preferred Reporting Items for Systematic Review and Meta-Analysis (PRISMA) guidelines [38]. A systematic search was conducted with the following six electronic databases from January 2010 to March 2021: Pubmed, Cochrane Library, Embase, Web of Science, the China National Knowledge Infrastructure (CNKI), and Wanfang Database. The studies in English and Chinese were all included. The following Mesh terms and the synonyms were used: “Sarcopenia”, “Muscular Atrophy”, “Aging”, “Aged”, “Frailty”, “Randomized Controlled Trials”, “Blind Method”, “Exercise”, “Exercise Therapy”, “Resistance Training”, “Endurance Training”, “Vibration”. We also used the terms “NOT (COPD, Cancer, Kidney Diseases)” (Full search strategy see Supplement 1).

### Study selection and data extraction

Two researchers independently screened the title and abstract of each retrieved study to exclude irrelevant studies. Repeated studies for the same exercise RCT were excluded as were reviews of animal studies. The full-text of each remaining study was systematically evaluated according to the inclusion and exclusion criteria. The bibliographic information for author, publication year, the characteristics of subjects (sample size, gender, mean age, and appendicular skeletal muscle mass/height^2^ [ASM/ht^2^]), details of the exercise interventions (duration, type, frequency, and intensity), and the outcome measurements for body composition, muscle strength, and physical performance were independently extracted by two researchers. If a study was a multiple-arm trial, only the data of relevant exercise groups were extracted. A summary of the study results was recorded in a standard table format developed for this study. If information was recorded differently by the two researchers, a third researcher discussed the difference until it was resolved.

### Quality assessment

Two researchers independently assessed the methodological quality of the studies using the Physiotherapy Evidence Database (PEDro) scale [39]. The following 11 characteristics were used to assess the quality of studies: Eligibility criteria, Random allocation, Concealed allocation, Baseline similar, Blinding (Subjects), Blinding (Therapists), Blinding (Assessors), Measure for > 85%, Intention-to-Treat Analysis, Group comparison and Point measures. Each characteristic was rated as 0 (not meeting the criteria) to 1 (meeting the criteria). A high total score indicated higher study quality. When differences in ratings occurred between two researchers, a third researcher discussed the problem until it was resolved.

Two researchers independently assessed the risk of bias using the Review Manager (RevMan 5.4; Cochrane, Lindon, UK). Among the myriad of biases [40, 41] the bias assessment included selection bias, performance bias, detection bias, attrition bias and reporting bias. Each study was assigned a bias category of low risk, unclear risk or high risk. Biases not evaluated in this study were listed as other potential biases and assigned a category of unclear risk. Differences in the identification of study biases between the two researchers was resolved by discussion with a third researcher. Following agreement of bias for each study, the percentage of bias categories was calculated.

We used the Grades of Recommendation, Assessment, Development and Evaluation (GRADE) assessment to classify the overall certainty of evidence across studies of the outcomes and absolutely reduce the risk by using GRADE profiler version 3.6 [42] . The evidence of the outcomes of studies will be divided into one of the four grades [43]: (a) High: We are very confident that the true effect lies close to that of the estimate of the effect and progressive study is unlikely to change this result; (b) Moderate: We are moderately confident in the effect estimate, and the true effect is likely to be close to the estimate of the effect, but there is a possibility that it is substantially different; (c) Low: We have limited confidence in the results. Further research is very likely to have an important impact on our confidence on the estimate of effect and is likely to change the estimate; (d) Very low: We have little confidence in the results. The results may differ greatly from the real values, and further research is likely to change the results.

Originally, the evidence quality of RCTs is generally “High”, and the following five factors will reduce the evidence quality to “Moderate”, “Low” and “Very low”: (a) Risk of bias [44]: without concealed allocation, without assessor blinded or other limitations; (b) Inconsistency [45]: excessive heterogeneity and thus inconsistency of results); (c) Indirectness [46]: indirect populations, interventions, controls and outcomes; (d) Imprecision [47]: relatively small simple size, wide confidence intervals, fewer studies; (e) Publication bias [48]: graphically in funnel plots or many potentially studies have not been published.

### Statistical analyses

Review Manager (RevMan 5.4; Cochrane, Lindon, UK) was used to analyze all data. The statistical heterogeneity for the outcome in included studies was assessed by the I^2^ statistic. The analytic model used was dependent on the presence of heterogeneity; the fixed-effects model used when I^2^ < 50% and random-effects model was used when I^2^ > 50%. To perform the meta-analysis, data with continuous outcomes were analyzed by the changes in the means and standard deviations (SD) of the outcome measurement. Weighted mean differences (WMD) and 95% confidence intervals (95% CI) were computed when the studies had the same measurement methods and units for the independent and dependent variables. Standardized mean differences (SMDs) and 95% CI were computed when the studies had different measurement methods and units for the independent and dependent variables. In this meta-analysis, no studies met the criteria required to present results as WMD and 95% CI. For studies where the mean and SD could not be extracted completely, the author was contacted in attempt to obtain the data. If the author could not be contacted, the study was excluded from analyses. *P* < 0.05 was considered as statistical significance.

## Results

### Study selection

**Fig. 1:**
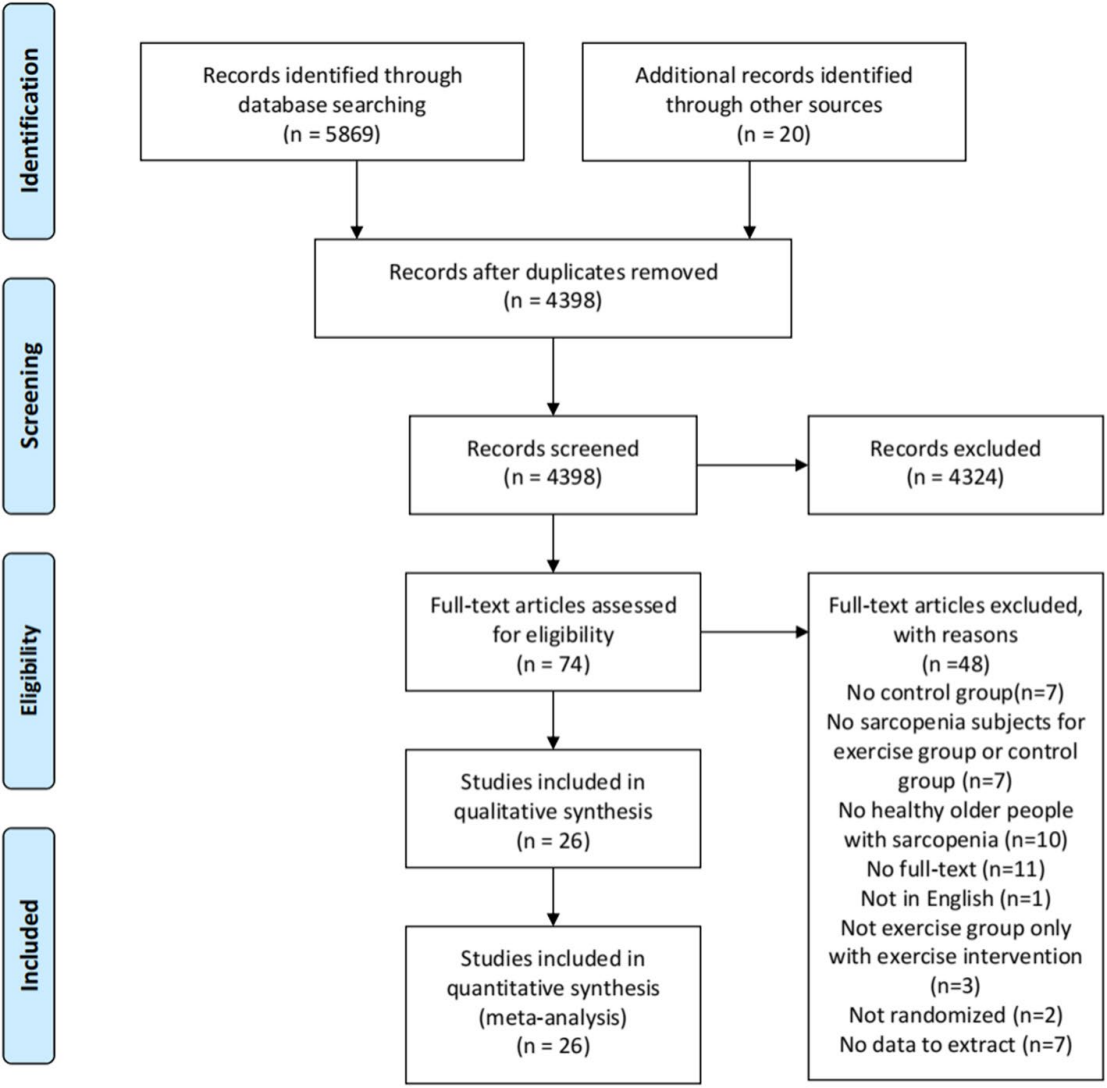
The flowchart for study screening and selection process according to the PRISMA guidelines

Figure 1 shows the flowchart for study screening and selection process according to the PRISMA guidelines. A total of 5889 records were retrieved, with 5730 retrieved using the keywords (Mesh terms and the synonyms) from Pubmed (*n* = 876), Cochrane Library (*n* = 815), Embase (*n* = 2436), Web of Science (*n* = 1603), the China National Knowledge Infrastructure (CNKI) (*n* = 107) and Wanfang Database (*n* = 32). Twenty studies were identified from the systematic reviews and other reviews retrieved in the search process. After removing duplicate studies, 4386 studies remained. The study titles and abstracts then were screened to apply the inclusion and exclusion criteria. This resulted in 4319 studies being removed for failing to meet the inclusion criteria. Of the remaining 74 studies, the full text was read to apply the exclusion criteria. The final sample size was 26 studies with full text for the systematic review and meta-analysis.

### Study characteristics

Tables 1, 2 and 3 shows the characteristics of the 26 studies to include the publication dates, sample size and populations studied by gender and age, sarcopenia diagnosis, intervention details of exercise mode, intensity, duration, and training movements, inclusion of a control group, and outcomes of the studies. Two studies included two exercise modes. Chen et al. [49] included RT and MT and Bellomo et al. [50] included RT and WBVT. The studies are included in the analysis for each of their exercise training modes.

**Table 1:** Characteristics of Included Studies with Resistance Training as an Exercise Training Mode

RT	Study	Sample size (ETG/CG)	M/F	Age	Sarcopenia diagnostics	Intervention				Control group	Outcome
						Mode	Training movement	Intensity	Duration Days/week (weeks)		
1	Bellomo et al. (2013) [43]	10/10	20/0	Average: 70.9 ± 5.2	MMI by DXA, < 2 standard deviations of a young reference population (CDC)	Weight machine	leg press and leg extension	W1–4: 3 sets/12 reps, 60–70% FMT; W5–8: 3 sets/10 reps, 75–80% FMT; W9–12: 3 sets/6–8 reps, 80–85% FMT	2(12)	Non-exercise	KES
2	Cebrià et al. (2018) [45]	11/17	7/21	82.6 ± 9.1/81.2 ± 5.4	ASMI by BIA, M: ≤0.93 kg/m^2^, F:≤0.57 kg/m^2^; GS, M: 0.95–0.66 m/s; F: 0.08–0.48 m/s (Tyrovolas)	Dumbbells, and ankle/wrist weights	UL: handgrip, wrist flexion/extension, forearm pronation/supination, elbow flexion/extension, shoulder flexion/extension /adduction/abduction; LL: ankle flexion/ extension, knee extension, and hip flexion/abduction/ adduction	10 sets/12 reps, 40–60% 1RM	3(12)	Non-exercise	ASMI; HGS, KES; GS
3	Chen et al. (2017) [42]	15/15	5/25	68.9 ± 4.4/68.6 ± 3.1	ASMI by BIA, M: ≤32.5%, F: ≤25.7% (Chung)	Weight machine	shoulder presses, bicep curls, triceps curls, bench presses, deadlifts, leg swings, squats, standing rows, unilateral rows, split front squats	3 sets/8–12 reps; 60–70% 1RM	2(8)	Non-exercise	ASMI, SMM; HGS, KES
4	Hamaguchi et al. (2017) [51]	7/8	0/15	60.4 ± 2.7/60.6 ± 2.3	SMI by DXA, < 6.12 kg/m^2^ (EWGSOP-2010)	Weighted vest	squats, front lunges, side lunges, calf raises, toe raises	8 sets/3 reps	2(6)	Non-exercise	SMI; HGS, KES
5	Liao et al. (2017) [52]	25/21	0/46	66.39 ± 4.49/68.42 ± 5.86	SMI by DXA, F: ≤7.15 kg/m^2^ (EWGSOP-2010)	Thera-band	seated chest press, seated row, seated shoulder press, Concentric–eccentric hip circumduction, leg press, leg curl	3 sets/10 reps; Intensity: Borg RPE 13	3(12)	Non-exercise	SMI; HGS, KES; GS, TUG, CS;
6	Liao et al. (2018) [53]	30/20	0/50	66.67 ± 4.54/68.32 ± 6.05	ASMI by BIA, M: ≤32.5%, F: ≤25.7% (Chung)	Thera-band	UL: seated chest press, seated row seated shoulder press; LL: knee extension, knee flexion, hip flexion, hip extension.	3 sets/10 reps; Intensity: Borg RPE 13	3(12)	Non-exercise	SMI, AMI; KES; GS, TUG, CS
7	Shao et al. (2020) [46]	41/30	45/26	69.33 ± 13.47/68.54 ± 10.62	ASMI by BIA, M: < 7 kg/m^2^, F: < 5.7 kg/m^2^; HGS, M: < 26 kg; F: < 18 kg; GS < 0.8 m/s (AWGS)	Weight machine	knee extension/flexion, chest press, shoulder press, and low back muscle	30 min/time	2(24)	Education (4 weeks/time)	SMI; HGS; GS
8	Vikberg et al. (2019) [47]	36/34	32/38	70.9 ± 0.28 /70.0 ± 0.29	ALMI by DXA, M: ≤7.29 kg/m^2^; F: ≤5.53 kg/m^2^ (EWGSOP-2010)	Body weight and suspension bands	Squats, Calf Raises, Chair Stands, Half Lunges, Biceps Rowing, Push-ups, and Bridge	W1: 2 sets/12 reps; W2–4: 3 sets/10 reps; W5–7: 4 sets/10 reps; W8–10: 4 sets/10 reps. Intensity: Borg RPE 10	3(10)	Non-exercise	ALMI; HGS; TUG, CS, SPPB, 4MWT

*RT* resistance training, *ETG* exercise training group, *CG* control group, *UL* upper limb, *LL* lower limb, *M* male, *F* female, *reps* repetitions, *1RM* one repetition maximum, *FNT* maximum theoretical force, *RPE* rated perceived exertion, *W* week, *DXA* Dual energy X-ray absorptiometry, *BIA* Bioelectrical impedance analysis, *MMI* muscle mass index (MMI = muscle mass/height^2^[MMI/m^2^]), *SMM* skeletal muscle mass, *SMI* skeletal muscle mass index (SMI = skeletal muscle mass/ height^2^ [SMI/m^2^]), *AMI* appendicular muscle mass index (AMI = appendicular muscle mass/height^2^ [AMM/m^2^]), *ASMI* appendicular skeletal muscle mass index (ASMI = appendicular skeletal muscle mass/height^2^ [ASM/m^2^]), *ALMI* appendicular lean muscle mass index (ALMI = appendicular lean muscle mass/ height^2^ [ALM/m^2^]), *AWGS* Asian Working Group for Sarcopenia, *EWGSOP* European Working Group on Sarcopenia in Older People, *HGS* hand grip strength, *KES* knee extension strength, *GS* gait speed, *TUG* Timed Up and Go, *CS* Chair stand, *SPPB* Short Physical Performance Battery, *4MWT* 4-m walk test

**Table 2:** Characteristics of Included Studies Whole Body Vibration Training as an Exercise Training Mode

VT	Study	Sample size (ETG/CG)	M/F	Age	Sarcopenia diagnostics	Intervention				Control group	Outcome
						Mode	Training movement	Intensity	Duration Days/week (weeks)		
1	Bellomo et al. (2013) [43]	10/10	20/0	70.9 ± 5.2	MMI by DXA, < 2 standard deviations of a young reference population (CDC)	Vibratory mechine	Received a focused vibratory stimulation; The stimulation was applied on the vastus medialis, vastus lateralis; rectus femoris muscles.	300 Hz	W1–8: 1 session /week; W9–12: 3 session s/week	Non-WBV	KES
2	Wei et al. (2016) [48]	20/20	12/28	75 ± 6/76 ± 6	SMI by BIA, M: < 8.87 kg/m^2^, F: < 6.42 kg/m^2^ (EWGSOP-2010)	WBV platform	Stood on the platform of a WBV machine without shoes, knees were kept at 60° of flexion, hands placed onto the rail in front for support	40 Hz, 4 mm; 4sets x 90s (6 min)	3(12)	Non-WBV	SMI; KES; TUG, CS, GS
3	Zhu et al. (2016) [54]	28/27	55/0	89.5 ± 4.4/87.5 ± 3.0	SMI by DXA: M: < 7.0 kg/m^2^, F: < 5.4 kg/m^2^ (AWGS)	WBV platform	stand on the WBV plate, hands clasping the WBV rope, with legs and knees slightly bent	12–16 Hz, 3–5 mm	5(8)	Non-WBV	SMM; HGS; GS, TUG, CS
4	Zhu et al. (2019) [55]	28/27	55/0	89.5 ± 4.4/87.5 ± 3.0	SMI by DXA, M: < 7.0 kg/m^2^, F: < 5.4 kg/m^2^; BIA, M: < 7.0 kg/m2, F: < 5.7 kg/m^2^ (AWGS)	WBV platform	stand on the WBV plate, with legs and knees slightly bent, hands clasping the WBV rope	12–16 Hz, 3–5 mm; 5 groups/time, 3 min/group.	5(8)	Non- WBV	MM; HGS, KES; TUG, CS
5	Wei et al. (2016) [44]	LG/CG: 20/20	12/28	78 ± 4 /76 ± 6	SMI by BIA, M: < 8.87 kg/m^2^, F: < 6.42 kg/m^2^ (EWGSOP-2010)	WBV platform	Stood barefoot with knee joint flexed at 60° on the platform of a whole-body WBV machine; hands holding onto the rail in front for support.	20 Hz × 720 s (12mins), 4 mm	3(12)	Non-WBV	SMI; KES
		MG/CG: 20/20	12/28	75 ± 6/76 ± 6				40 Hz × 360 s (6mins), 4 mm			
		HG/CG: 20/20	10/30	74 ± 5/76 ± 6				60 Hz × 240 s (4mins), 4 mm			
6	Wei et al. (2017) [49]	LG/CG: 20/20	12/28	78 ± 4/76 ± 6	SMI by BIA, M: < 8.87 kg/m^2^, F: < 6.42 kg/m^2^ (EWGSOP-2010)	WBV platform	stood barefoot with knee joint flexed at 60° on the platform of the WBV machine with hands holding onto the rail in front for support	20 Hz × 720 s, 4 mm	3(12)	Non-WBV	SMI; TUG, CS, GS
		MG/CG: 20/20	12/28	75 ± 6/76 ± 6				40 Hz × 360 s, 4 mm			
		HG/CG: 20/20	10/30	74 ± 5/76 ± 6				60 Hz × 240 s, 4 mm			

*VT* vibration training, *ETG* exercise training group, *CG* control group, *WBV* whole body vibration training, *M* male, *F* female, *W* week, *DXA* Dual energy X-ray absorptiometry, *BIA* Bioelectrical impedance analysis, *MM* muscle mass, *MMI* muscle mass index (MMI = muscle mass/height^2^ [MMI/m^2^]), *SMM* skeletal muscle mass, *SMI* skeletal muscle mass index (SMI = skeletal muscle mass/ height^2^ [SMI/m^2^]), *AWGS* Asian Working Group for Sarcopenia, *HGS* hand grip strength, *KES* knee extension strength, *GS* gait speed, *TUG* Timed Up and Go, *CS* Chair stand, *LG* low frequency and long exercise time group, *MG* medium frequency and medium exercise time group, *HG* high frequency and short exercise time group

**Table 3:** Characteristics of Included Studies Mixed Training as an Exercise Training Mode

MT	Study	Sample size (ETG/CG)	M/F	Age	Sarcopenia diagnostics	Intervention				Control group	Outcome
						Mode	Training movement	Intensity	DurationDays/week (weeks)		
1	Bagheri et al. (2020) [56]	10/10	20/0	63.8 ± 3.6/65 ± 3.9	HGS < 26–30 kg, GS < 0.8 m/s, BMI at least two standard deviations below the average young population (EWGSOP-2010)	Resistance machine	1.RT: leg extension, leg curl, bench press, lat pulldown, lateral raise, abdominal crunch.	Beginning: 8–16 reps, 40% 1RM; Progressed to: 75% 1RM	3(8)	Non-exercise	SMM; KES
						Fixed-speed bike	2.ET	Beginning: 55% maxHR, 15 min;Progressed to: 70% maxHR, 30 min			
2	Chen et al. (2017) [42]	15/15	5/25	68.5 ± 2.7/68.6 ± 3.1	ASMI by BIA, M: ≤32.5%, F: ≤25.7% (Chung)	Weight machine	1.RT: shoulder presses, bicep curls, triceps curls, bench presses, deadlifts, leg swings, squats, standing rows, unilateral rows, and split front squats	3 sets/8–12 reps;60–70% 1RM	1(8)	Non-exercise;	SMM; HGS, KES
						/	2.AT: a combination of dance steps such as stepping on the spot, knee lifts, high knee running, rowing arm swings, arm swings, twist steps, arm raises, squats, V steps, mambo steps, diamond steps, and point step jumps;	/			
3	Hassan et al. (2015) [57]	20/21	Not identified	85.9 ± 7.5	SMI by BIA, M: < 8.87 kg/m^2^, F: < 6.42 kg/m^2^ HGS, M: < 30 kg, F: < 20 kg; GS, < 0.8 m/s (EWGSOP2010)	Air-pneumatic equipment	1.RT: elbow and shoulder extension, leg press, knee extension and flexion, hip abduction and adduction, abdominal curl and back extension	2–3 sets/10–15 times;Intensity: Borg RPE 12–14	2(24)	Non-exercise	SMI; HGS; GS
							2.Balance exercises: heel and toe raise, varied directional quick stepping, reaching, single leg standing, static balance, heel to toe walking and complex cross over stepping activities.	3 sets/10 reps →3 sets/15 reps			
4	Kim et al. (2012) [58]	39/39	0/78	79.0 ± 2.9/78.7 ± 2.8	1) ASMI by BIA < 6.42 kg/m^2^ and KES < 1.01 Nm/kg; 2) ASMI by BIA < 6.42 kg/m^2^ and GS < 1.22 m/s; 3) BMI < 22.0 kg/m, KES < 1.01 Nm/kg; 4) BMI < 22.0 kg/m^2^ and GS < 1.22 m/s (Kim 2012/2013)	Resistance band or ankle weight	1.RT: (1) Chair exercise: ①Repetitions of toe raises, heel raises, knee lifts, knee extensions, and others were performed while seated on a chair; ②Hip flexions, lateral leg raises, and repetitions of other exercises while standing upright behind the chair and holding the back of the chair for stability. (2) Ankle-weight exercise: seated knee flexion, extension, standing knee flexion extensions	1 set/8 reps; Intensity: Borg RPE 12–14	2(12)	Education (1 month/time, 3 times)	MM, AMM; KES; GS
							2.Balance and gait training: raising the toes during the forward swing of the leg, kicking off the floor with the ball of the foot, walking with directional changes, gait pattern variations.	Weights of 0.50, 0.75, 1.00, and 1.50 kg			
5	Kim et al. (2013) [59]	32/32	0/64	79.6 ± 4.2/80.2 ± 5.6	1) ASMI < 6.42 kg/m^2^ and KES < 1.01 Nm/kg; 2) ASMI < 6.42 kg/m^2^ and GS < 1.22 m/s; 3) BMI < 22.0 kg/m, KES < 1.01 Nm/kg; 4) BMI < 22.0 kg/m^2^ and GS < 1.22 m/s. (Kim 2012/2013)	Resistance band and ankle weight	1.RT: focused on hip extensors and adductors, knee flexors and extensors, ankle dorsi, plantar flexors.	1 set/8 reps→ gradually increased to 2 sets/10 reps	2(12)	Non-exercise	MM, ASM; HGS, KES; GS, TUG
						/	2.Gait and balance training: toe elevation of the forward leg, heel elevation of the rear limb; one-leg stands, tandem stand, tandem walking	/			
6	Kim et al. (2016) [60]	35/34	0/69	81.4 ± 4.3/81.1 ± 5.1	SMI by DXA: < 5.67 kg/m^2^; HGS < 17.0 kg; GS < 1.0 m/s (Kim 2016)	Weight machine; Resistance band; Hydraulic exercise machine	1.RT: (1) Chair exercise: toe raise, heel raises, knee lifts, knee extensions; hip flexions, lateral leg raises, standing upright behind the chair and holding the back; (2) Resistance band exercise: double-arm pulldowns, bicep curls, leg extensions, hip flexions; (3) Machine: seated row, leg press, abduction, leg extension, abdominal crunch machines	1–3 sets/10 reps	2(12)	Education (2 weeks/time, 6 times)	MM, AMM, ASM; HGS, KES; GS
						**/**	2.AT	1 set/10 reps→3 sets/10 reps			
7	Makizako et al. (2020) [33]	36/36	21/51	74.1 ± 6.6/75.8 ± 7.3	ASMI by BIA, M: < 7.0 kg/m^2^, F: < 5.7 kg/m^2^ (AWGS)	Resistance band	1.RT: knee extension, hip flexion (knee raises), hip internal rotation, elbow flexion and shoulder abduction, elbow flexion and trunk rotation, hip extension, knee flexion, hip abduction, and squat.	5 resistance levels/10 reps; Intensity: Borg RPE 12–14	12 weeks	Education (60 min, 1 time)	ASMI; HGS; GS, CS, TUG
						/	2.Balance and AT: tandem stand, heel-up stand, one-leg stand, weight shifts, and stepping; anterior-posterior or lateral stepping repetitions for 6 min.	/			
8	Maruya et al. (2016) [50]	26/14	23/29	69.2 ± 5.6/68.5 ± 6.2	SMI by BIA, M: < 7.0 kg/m^2^, F: < 5.7 kg/m^2^ (AWGS)	Weight	1.RT: (1) Squats: a 6-s movement time, and then to slowly return to their standing position; (2) Single-leg standing: a single-leg standing posture for 1 min, using light touch on stable desk or chair; (3) Heel raises	3 sets/6 reps; 3 sets; 3 sets/20 reps	6 months	Non-exercise	SMI; HGS, KES; GS, SPPB
						/	2.AT: walking	20–30 min/day			
9	Moghadam et al. (2020) [61]	10/10	20/0	64.1 ± 3.3/65 ± 3.9	HGS < 26–30 kg, GS < 0.8 m/s, SMI at least two standard deviations below the average young population (EWGSOP-2010)	Resistance machine	1.RT: leg extension, leg curl, bench press, lat pulldown, lateral raise, and abdominal crunch	Beginning: 2 sets/16–18 reps, 40% 1RM; Progressed to: 3 sets/8–10 reps, 75% 1RM	3(8)	Non-exercise	KES
						fixed-speed cycle ergometer	2.ET	Beginning: 55% of maxHR, 15 min; Progressed to: 70% maxHR, 30 min			
10	Park et al. (2017) [62]	25/25	0/50	73.5 ± 7.1/74.7 ± 5.1	ASM/weight <25.1% (Park)	/	1.AT: sideways, backward, and forward walking, slow and fast indoor walking;	W1–12: 13–15 RPE; W13–24: ≤15 RPE	5(24)	Education (2 times)	ASM; HGS; CS, GS
						Thera-Band	2.RT: elbow flexion, wrist flexion, shoulder flexion, lateral raise, front raise, chest press, reverse flies, side band, dead lift, squat, leg press, ankle plantar flexion.	W1–12: 8–11 reps/set; W13–24: 12–15 reps/set	3(24)		
11	Shahar et al. (2013) [63]	19/16	Not identified	69.74 ± 5.46/67.25 ± 5.48	SMI by BIA, M: < 10.75 kg/m^2^, F: < 6.75 kg/m^2^ (NHANES III)		1.AT: walking and jogging on the spot; walking forwards, backwards, sideways; arm movements; raising the head, moving it in circularly; moving the trunk in four directions; and pushing knee forwards and backwards.	/	2(12)	Relaxation (2 weeks/ time, 6 times)	MM; HGS; CS, TUG, 6MWT
						Elastic band	2.RT: shoulder abduction; shoulder flexion; triceps extension; overhead press, side lateral extension; a standing double biceps curl; leg extension; foot extension; plantar flexion.				
						/	3.Balance exercises: stand on their heels; stand on one leg with the other leg on the ground or lifted behind or aside; stand on one leg while lifting up the opposite hand; and to walk foot by foot with fingers touching the foot from the back.				
12	Tsekoura et al. (2018) [23]	18/18	4/32	74.56 ± 6.04/72.89 ± 8.31	SMI by BIA, M: < 7.23 kg/m^2^, F: < 5.67 kg/m^2^ (EWGSOP-2010)	Weight	1.AT: walking	30–35 min/time	3(12)	Education	SMI; HGS, KES; GS, TUG, CS
							2.RT: knee extensor-flexor, hip abductor-extension, ankle plantar flexors- dorsi flexors, wall push up, seated bicep curl, seated triceps extension, seated lateral shoulder raises, seated abdominal crunches	W1–4: 1 set/8 reps; Intensity: Borg RPE 10–11 (Easy) W5–8: 2 sets/10 reps; Intensity: Borg RPE 10–12 (Easy) W9–12: 2 sets/12reps; Intensity: Borg RPE 12 (Medium)	2(12)		
							3.Balance and gait training: walking and turning around, walking- backward tandem-heel-toe- heel to toe-sideways, one leg stand, tandem stance	/			
13	Vasconcelos et al. (2016) [64]	14/14	0/28	72 ± 4.6/72 ± 3.6	BMI ≥30 kg/m^2^, HGS ≤21 kg (Fried)	/	1. stretching exercise: remained the same, 60 s in each leg for posterior, anterior, lateral, and medial muscles of hips and knees	Using the modified Borg Scale	2(10)	Non-exercise	KES; GS, SPPB
						Weight	2.RT: (1) W1–4: a low speed concentric and eccentric movements) → the high-speed (focusing on muscle power); (2) W5–6: perform the concentric movements of exercises “as fast as possible”; (3)W7–10: concentric and eccentric movements in high speeds	W1–2: (1) Knee exercise: 2 sets/12 reps, 50% 1RM; (2) Hip exercises: 2 sets/8 reps; (3) Mini-squats, against the wall: 2 sets/10 reps W3–4: (1) Knee exercise: 2 sets/12 reps, 75% 1RM; (2) Hip exercises: 2 sets/8 reps, 2 kg; (3) Mini-squats: 2 sets/10 reps			
								W5–6: (1) Knee exercise: 2 sets/12 reps, 40% 1RM; (2) Hip exercises: 2 sets/8 reps, 2 kg; (3) Mini-squats: 2 sets/10 reps			
								W7–8: (1) Knee exercise: 2 sets/12 reps, 60% 1RM; (2) Hip exercises: 2 sets/8 reps, 3 kg; (3) Mini-squats: 2 sets/10 reps W9–10: (1) Knee exercise: 3 sets/12 reps, 60% 1RM; (2) Hip exercises: 3 sets/8 reps, 3 kg; (3) Mini-squats: 3sets/10 reps			
14	Zhu et al. (2019) [65]	40/37	19/58	74.5 ± 7.1/72.2 ± 6.6	SMI by BIA, M: < 7.0 kg/m^2^, F: < 5.7 kg/m^2^ (AWGS)	Thera-Band	1.RT: chest, back, biceps, triceps; knee extension/flexion, heel raises, side leg lifts)	6 sets/6–8 reps; 40% 1RM	2(12)	Non-exercise	ASMI, AMM; HGS; GS, CS
						/	2.AT	/			

*MT* mixed training, *ETG* exercise training group, *CG* control group, *M* male, *F* female, *reps* repetitions, *1RM* one repetition maximum, *RPE* rated perceived exertion, *maxHR* maximum Heart Rate, *W* week, *DXA* Dual energy X-ray absorptiometry, *BIA* Bioelectrical impedance analysis, *BMI* body mass index, *MM* muscle mass, *SMM* skeletal muscle mass, *SMI* skeletal muscle mass index (SMI = skeletal muscle mass/ height^2^ [SMI/m^2^]), *AMM* appendicular muscle mass, *ASM* appendicular skeletal muscle mass, *ASMI* appendicular skeletal muscle mass index (ASMI = appendicular skeletal muscle mass/height^2^ [ASM/m^2^]), *AWGS* Asian Working Group for Sarcopenia, *EWGSOP* European Working Group on Sarcopenia in Older People, *HGS* hand grip strength, *KES* knee extension strength, *GS* gait speed, *TUG* Timed Up and Go, *CS* Chair stand, *SPPB* Short Physical Performance Battery, *6MWT* 6-m walk test, *RT* resistance training; *AT* aerobic training, *ET* endurance training

#### Publication dates and languages

The 26 studies were published from 2012 to 2020 with four in 2020, three in 2019, three in 2018, five in 2017, six in 2016, one in 2015, three in 2013, and one in 2012. Two studies were written in Chinese and the rest were written in English.

#### Sample size and populations studied

A total of 1191 older people with sarcopenia were enrolled in the studies with 613 in exercise groups and 578 in the control groups. The sample sizes in the exercise groups ranged from seven to 41 subjects and the control groups ranged from eight to 39 subjects. The mean age of the subjects ranged from 60.6 ± 2.3 to 89.5 ± 4.4 [65]. 11 studies [16, 23, 33, 49, 51, 52, 54–56, 61, 65] included both genders, five [50, 53, 58–60] included only males, eight [57, 62–64, 66–69] included only females, and two studies [70, 71] did not identify the genders.

#### Sarcopenia diagnosis

Six studies [16, 33, 52, 53, 55, 59] diagnosed sarcopenia using the criteria from the Asian Working Group for Sarcopenia (AWGS), seven studies [23, 56, 58, 60, 62, 64, 70] used the criteria from the European Working Group on Sarcopenia in Older People (EWGSOP), and the remaining 13 studies [49–51, 54, 57, 61, 63, 65–69, 71] did not describe their diagnostic criteria in detail, only that they used specific index values. Table 4 shows the different sarcopenia diagnostic criteria used in all included studies.

**Table 4:** Different indicators and cut-off points in defining sarcopenia

Diagnosis Criteria	Target district	Cut-off points		
		Muscle mass	Muscle strength	Physical performance
AWGS [72]	countries from Asia	ASMI by DXA: (M: < 7.0 kg/m^2^, F: < 5.4 kg/m^2^); Or ASMI by BIA: (M: < 7.0 kg/m^2^, F: < 5.7 kg/m^2^)	HGS: (M:< 28 kg, F:< 18 kg)	GS: < 1.0 m/s; Or 5-STS ≥12 s; Or SPPB: ≤9
EWGSOP-2010 [73]	countries from Europe	ASMI by DXA: (M: < 7.23 kg/m^2^, F: < 5.67 kg/m^2^); or ASMI by BIA: (M: < 8.87 kg/m^2^, F: < 6.42 kg/m^2^)	HGS: (M: < 30 kg, F: < 20 kg)	GS: < 1.0 m/s; or SPPB: ≤8
CDC [74]	New Mexico	ASM* < 2 standard deviations of a young reference population	/	/
National Center for Health Statistics [75]	NHANES III	SMI by BIA, M: < 10.75 kg/m^2^, F: < 6.75 kg/m^2^	/	/
Park [62]	Busan City, South Korea	ASM2/weight <25.1%	/	/
Chung [76]	Korea	ASMI2 by BIA, M: ≤32.5%, F: ≤25.7%	/	/
Kim 2012/2013 [58, 59]	Tokyo	ASMI3 by BIA < 6.42 kg/m^2^; BMI < 22.0 kg/m	KES < 1.01 Nm/kg	GS < 1.22 m/s
Kim 2016 [60]	Tokyo	SMI by DXA < 5.67 kg/m^2^	HGS < 17.0 kg	GS < 1.0 m/s
Tyrovolas [77]	countries from Asia, Africa, Europe, and Latin America	ASMI4 by BIA, M: ≤0.93 kg/m^2^, F: ≤0.57 kg/m^2^	HGS: (M: < 30 kg, F: < 20 kg)	GS: (M: 0.95–0.66 m/s; F:0.08–0.48 m/s)
Fried [78]	countries from African American	baseline: >10lbs lost unintentionally in prior year	HGS: lowest 20% (by gender, body mass index)	walking time/15 ft: slowest 20% (by gender, height)

*AWGS* Asian Working Group for Sarcopenia, *EWGSOP* European Working Group on Sarcopenia in Older People, *CDC* Centers for Disease Control and Prevention, *NHANES III* Third National Health and Nutrition Examination Survey, *M* male, *F* female, *ASMI* appendicular skeletal muscle mass index (ASMI = appendicular skeletal muscle mass/height^2^[ASM/m^2^]), *ASMI2* = appendicular skeletal muscle mass/weight^2^*100% [ASM/kg^2^], *ASMI3* appendicular skeletal muscle mass index (ASMI = appendicular skeletal muscle mass/height^2^[ASM/m^2^]), *ASMI4* = [0.244(weight) + 7.8(height) + 6.6(sex)-0.098(age) + race-3.3]/body mass index (ASM/BMI), *SMI* skeletal muscle index; ASM* = 0.2487(weight) + 0.0483(height)-0.1584(hip circumference) + 0.0732 (grip strength) + 2.5843(sex) + 5.8828, *ASM2* appendicular skeletal muscle mass, *DXA* Dual energy X-ray absorptiometry, *BIA* Bioelectrical impedance analysis, *HGS* handgrip strength, *KES* knee extension strength, *GS* gait speed, *5-STS* 5-chair sit to stand test, *SPPB* Short Physical Performance Battery, *TUG* Timed Up and Go

#### Intervention details

For the 20 RT studies [16, 23, 49, 50, 54–58, 60, 63, 66, 67, 69],the following training movements were used: six studies [49, 50, 55, 58, 60, 67] used weight machines, four studies [16, 23, 56, 69] used individual body weights, one study used dumbbells [54], three studies [54, 63, 66] used ankle/wrist weights, ten studies [33, 52, 56, 57, 63, 64, 66–68, 71] used bands, and one study [62] used weighted vests.

Twelve studies [23, 33, 49, 52, 54, 55, 57, 64, 67, 68, 70, 71] focused on the muscles of upper and lower limbs and nine studies [16, 50, 56, 58, 60, 62, 63, 66, 69] focused on the muscles of lower limbs. The exercise intensities were distributed as follows: six studies [49, 52, 54, 58, 60, 69] ranged from 60 to 80% of 1 repetition maximum (RM), one study was 60–85% of maximum theoretical force [50], ten studies [23, 33, 56, 57, 63, 64, 66–68, 70] use the Borg CR-10 scale and four studies [16, 55, 62, 71] did not describe the intensity of the training. The duration of the exercise sessions lasted 20 to 60 min, the training frequency varied from 1 to 5 times per week and the length of the training period varied from 6 weeks to 6 months.

For WBVT, all six studies [50, 51, 53, 59, 61, 65] used a WBV machine to apply the training. The frequency of WBVT ranged from 12 to 60 Hz in five studies [51, 53, 59, 61, 65] with one study [50] using a time-modulated sinusoidal signal up to 300 Hz. The duration of the exercise sessions lasted of 15 to 40 min, the training frequency varied from 3 to 5 times per week, and the length of training period varied from 3 to 8 months.

For MT, all 14 studies [16, 23, 33, 49, 52, 58, 60, 63, 66–71] used a comprehensive progressive exercise program that included resistance and balance training (one study [70]), resistance and endurance training (two studies [58, 60]), resistance, balance and gait training (two studies [63, 66]), resistance, balance and aerobic training (two studies [33, 71]), resistance, balance, aerobic and gait training (one study [23]), resistance and aerobic training (five studies [16, 49, 52, 67, 68]), and resistance training and stretching (one study [69]). The exercise sessions lasted 60 to 90 min, the training frequency varied from 1 to 5 times per week, and the length of training period varied from 8 weeks to 6 months.

#### Control group

Nineteen studies [16, 49–54, 56–65, 69, 70] required subjects to maintain their usual daily lifestyles without any exercise interventions, subjects in six studies [23, 33, 55, 66–68] received an educational course and subjects in one study [71] received a relaxation exercise program.

#### Outcome measures

Some studies measured outcomes that were not analyzed in this study. Among the studies with outcomes of interest, 17 studies [16, 23, 49, 50, 54, 57, 59–67, 69] measured muscle strength by KES, 11 studies [23, 33, 51, 53, 56, 57, 59, 61, 63, 64, 71] measured TUG time, 17 studies [16, 23, 33, 51–55, 57, 61, 63, 64, 66–70] measured GS, 11 studies [23, 33, 52, 53, 56, 57, 59, 61, 64, 68, 71] measured CS times.

### Study quality

The PEDro scores of each study for the quality assessment are shown in Table 5. The score ranges from 0 to 10 with a mean quality score of 6.36. Among the studies, four studies scored 8 points, eight studies scored 7 points, eleven studies scored 6 points and three studies scored 5 points. The quality assessment of the study consisted of 11 criteria: 25 studies reported the random allocation, and only one study performed non-randomized controlled trial. The baseline was similar in all studies. Seventeen studies [23, 33, 51–57, 59, 61, 63–67, 69] reported a concealed allocation. For blinding, three studies [33, 64, 70] used subject blinding, five studies [54, 57, 63, 64, 66] used therapists blinding and 11 studies [49, 50, 52, 54, 56, 62–64, 66, 68, 69] used assessor blinding. Twenty-three studies [16, 23, 33, 50–53, 55–62, 64–71] reported > 85% of the subjects performing at least one primary outcome measure, ten studies [23, 33, 53, 55, 58–60, 63, 68, 71] reported the data from intention-to-treat analysis, 25 studies [23, 33, 52, 54, 56–60, 62–64, 66–70] performed group comparison and 23 studies [23, 33, 52, 54, 56–63, 66–70] performed point measures.

**Table 5:** The PEDro Scores of Included Studies for The Quality Assessment

study	Eligibility criteria	Random allocation	Concealed allocation	Baseline similar	Blinding (subject)	Blinding (therapists)	Blinding (Assessor)	Measure for>85%	Intention-to-Treat Analysis	Group comparison	Point measures	Total score (0–10)
Bagheri et al. (2020) [56]	Yes	1	0	1	0	0	0	1	1	1	1	6
Bellomo et al. (2013) [43]	Yes	1	0	1	0	0	1	1	0	0	1	5
Cebrià et al. (2018) [45]	Yes	1	1	1	0	1	1	0	0	1	1	7
Chen et al. (2017) [42]	Yes	1	0	1	0	0	1	0	0	1	1	5
Hamaguchi et al. (2017) [51]	Yes	1	0	1	0	0	1	1	0	1	1	6
Hassan et al. (2015) [57]	Yes	1	0	1	1	0	0	1	0	1	1	6
Kim et al. (2012) [58]	Yes	1	1	1	0	1	1	1	0	1	1	8
Kim et al. (2013) [59]	Yes	1	1	1	0	1	1	0	1	1	1	8
Kim et al. (2016) [60]	Yes	1	1	1	0	0	0	1	0	1	1	6
Liao et al. (2017) [52]	Yes	1	1	1	1	1	1	1	0	1	0	8
Liao et al. (2018) [53]	Yes	1	1	1	0	1	0	1	0	1	1	7
Makizako et al. (2020) [33]	Yes	1	1	1	1	0	0	1	1	1	1	8
Maruya et al. (2016) [50]	Yes	1	0	1	0	0	0	1	0	1	1	5
Moghadam et al. (2020) [61]	Yes	1	0	1	0	0	0	1	1	1	1	6
Wei et al. (2016) [48]	Yes	1	1	1	0	0	0	1	0	1	1	6
Park et al. (2017) [62]	Yes	1	0	1	0	0	1	1	1	1	1	7
Shahar et al. (2013) [63]	Yes	0	0	1	0	0	0	1	1	1	1	6
Shao et al. (2020) [46]	Yes	1	1	1	0	0	0	1	1	1	0	6
Tsekoura et al. (2018) [23]	Yes	1	1	1	0	0	0	1	1	1	1	7
Vasconcelos et al. (2016) [64]	Yes	1	1	1	0	0	1	1	0	1	1	7
Vikberg et al. (2019) [47]	Yes	1	1	1	0	0	1	1	0	1	1	7
Wei et al. (2016) [44]	Yes	1	1	1	0	0	0	1	0	1	1	6
Wei et al. (2017) [49]	Yes	1	1	1	0	0	0	1	0	1	1	6
Zhu et al. (2019) [65]	Yes	1	1	1	0	0	1	1	0	1	1	7
Zhu et al. (2016) [54]	Yes	1	1	1	0	0	0	1	1	1	0	6
Zhu et al. (2019) [55]	Yes	1	1	1	0	0	0	1	1	1	1	7

*PEDro* Physiotherapy Evidence Database; 1: met the criteria; 0: not met the criteria

**Fig. 2:**
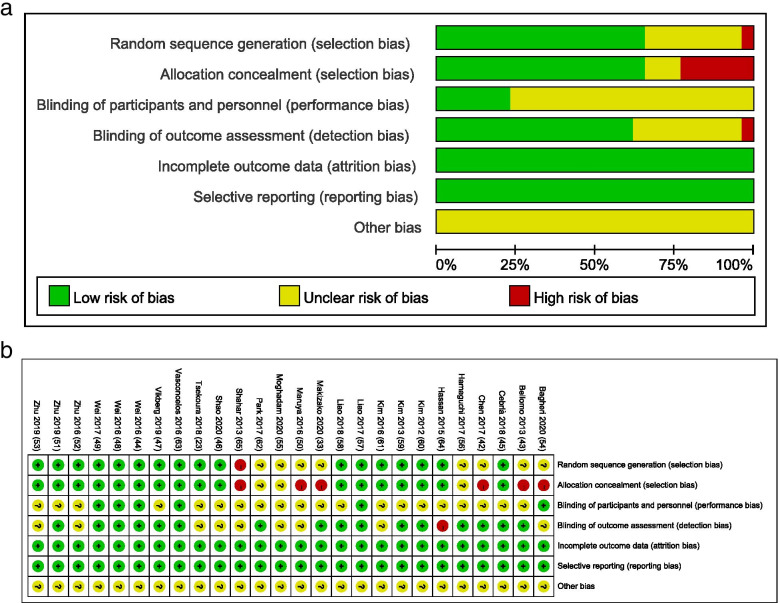
Assessment of risk of bias based on the Cochrane risk-of-bias tool. **a** Percent of studies with categories for risk of bias; **b** Summary for the risk of bias in each study

Details about the risk of bias of the included studies are shown in Fig. 2. Figure 2a is a plot of the distribution of studies at low-, unclear-, or high risk of bias based on the Cochrane risk-of-bias tool and Fig. 2b is a summary table for risks of bias in each study. For the random sequence generation assessment, the risk of bias was unclear in eight studies [16, 33, 49, 50, 58, 60, 62, 68] and the risk of bias was high in one study [71]. For the allocation concealment assessment, the risk of bias was unclear in three studies [60, 62, 68] and the risk of bias was high in six studies [16, 33, 49, 50, 58, 71]. For the participants and personnel assessment, the risk of bias was low in six studies [51, 58, 61, 64, 65, 69]. For the blinding of outcomes assessment, the risk of bias was unclear in nine studies [16, 23, 53, 55, 58–60, 67, 71] and the risk of bias was high in one study [70]. There were no unclear or high risk of bias observed for the incomplete outcome data assessment and the selective reporting assessment. The other bias risk items were not identified and were rated as unclear for all 26 studies.

The overall certainty of evidence across studies of the outcomes was performed in Table 6. After rating overall studies by the GRADE assessment, we found that the level of evidence was generally low. The possible reasons for this result are as follows: included assessor-blinding, concealed allocation, heterogeneity, length of follow-up and etc. Using the GRADE criteria, all these RCTs and non-randomised interventional studies showed a low level of evidence in KES as an indicator for muscle strength, and a low level of evidence in physical performance (TUG times, GS and CS times).

**Table 6:** The GRADE assessment for the overall certainty of evidence across studies

Quality assessment							Number of patients		Effect		Quality	Importance
Number of studies	Design	Risk of bias	Inconsistency	Indirectness	Imprecision	Other considerations	Muscle strength	Control	Relative (95% CI)	Absolute		
**KES subgroup (follow-up 0–24 weeks; Better indicated by lower values)**												
17	randomised trials	serious^a^	serious (I^2^ = 75)	no serious indirectness	no serious imprecision	none	415	394	–	SMD 0.86 higher (0.55 to 1.16 higher)	⊕ ⊕ OO LOW	CRITICAL
**TUG subgroup (follow-up 0–24 weeks; Better indicated by lower values)**												
9	randomised trials	serious^b^	serious (I^2^ = 60)	no serious indirectness	no serious imprecision	none	279	264	–	SMD 0.66 lower (0.94 to 0.38 lower)	⊕ ⊕ OO LOW	CRITICAL
**GS subgroup (follow-up 0–24 weeks; Better indicated by lower values)**												
17	randomised trials	serious^c^	serious (I^2^ = 87)	no serious indirectness	no serious imprecision	none	500	465	–	SMD 0.82 higher (0.43 to 1.21 higher)	⊕ ⊕ OO LOW	CRITICAL
**CS subgroup (follow-up 0–24 weeks; Better indicated by lower values)**												
10	randomised trials	serious^d^	serious (I^2^ = 87)	no serious indirectness	no serious imprecision	none	313	298	–	SMD 0.11 higher (0.36 lower to 0.57 higher)	⊕ ⊕ OO LOW	–

*KES* knee extension strength, *TUG* timed up and go, *GS* gait speed, *CS* chair stand

^a^ Of the 17 studies, 6 not performed concealed allocation and 9 were not blinded for assessors

^b^ Of the 9 studies, 6 were not blinded for assessors. ^c^ Of the 17 studies, 10 were not blinded for assessors. ^d^ Of the 10 studies, 6 were not blinded for assessors

### Outcomes

#### Effects of different exercise training modes for sarcopenia on KES

**Fig. 3:**
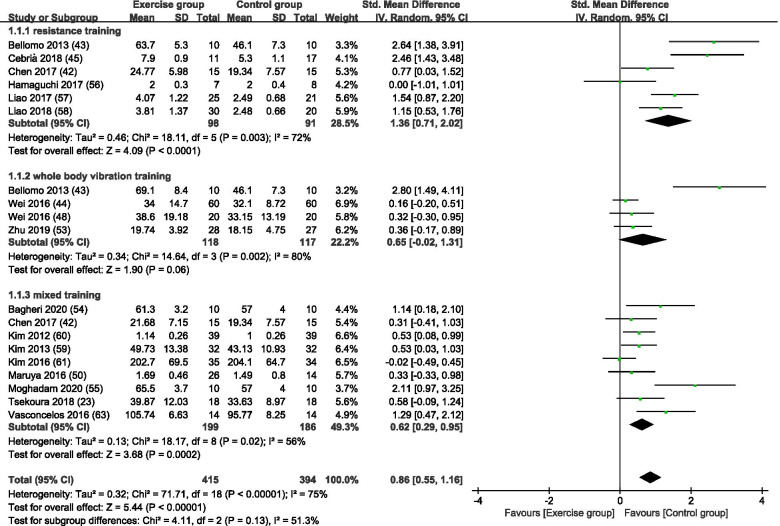
A forest plot of the subgroup analyses of 17 studies with KES as an outcome for sarcopenia based on different exercise training modes

Figure 3 is a forest plot of the subgroup analyses of 17 [16, 23, 49, 50, 54, 57–67, 69] studies with KES as an outcome for sarcopenia based on different exercise training modes. Collectively, 415 subjects were in exercise group and 394 subjects were in control group. Among subgroup analysis of the exercise modes, 6 studies [49, 50, 54, 57, 62, 64] used RT, 4 studies [50, 59, 61, 65] used WBVT training and 9 studies [16, 23, 49, 58, 60, 63, 66, 67, 69] used MT. With all exercise modes combined, the exercise group showed a significant increase in KES scores compared with the control group (SMD = 0.86, 95% CI: 0.55 to 1.16, *p* < 0.00001, I^2^ = 75%). In subgroup analysis, RT and MT resulted in significant increases in KES scores compared to the control group (RT, SMD = 1.36, 95% CI: 0.71 to 2.02, *p* < 0.0001, I^2^ = 72%; MT, SMD = 0.62, 95% CI: 0.29 to 0.95, *p* = 0.0002, I^2^ = 56%). WBVT resulted in no significant difference in KES scores between the exercise and control groups (SMD = 0.65, 95% CI: − 0.02 to 1.31, *p* = 0.06, I^2^ = 80%).

#### Effects of different exercise training modes for sarcopenia on physical performance

Nineteen studies [16, 23, 33, 54–57, 61, 63, 64, 66–71] assessed the effects of exercise training on the three main outcomes of TUG, GS, and CS.

**Fig. 4:**
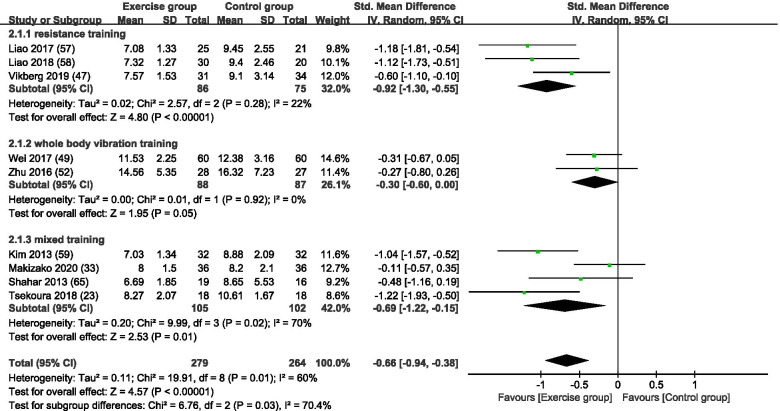
A forest plot of the subgroup analyses of 9 studies with TUG as an outcome for sarcopenia based on different exercise training modes

Figure 4 is a forest plot of the subgroup analyses of 9 studies (3 RT [56, 57, 64], 2 WBVT [51, 53], and 4 MT [23, 33, 63, 71]) with TUG as an outcome for sarcopenia based on different exercise training modes. Collectively, 279 subjects were in the exercise group and 264 subjects were in the control group. With all exercise modes combined, the exercise group showed a significant increase in TUG times compared with the control group (SMD = -0.66, 95% CI: − 0.94 to − 0.38, *p* < 0.00001, I^2^ = 60%). In subgroup analysis, all training modes showed a significant increase in TUG times compared with the control group (RT, SMD = -0.92, 95% CI: − 1.30 to − 0.55, *p* < 0.00001, I^2^ = 22%; WBVT, SMD = -0.30, 95% CI: − 0.60 to 0.00, *p* = 0.05, I^2^ = 0%; and MT, SMD = -0.69, 95% CI: − 1.22 to − 0.15, *p* = 0.01, I^2^ = 70%).

**Fig. 5:**
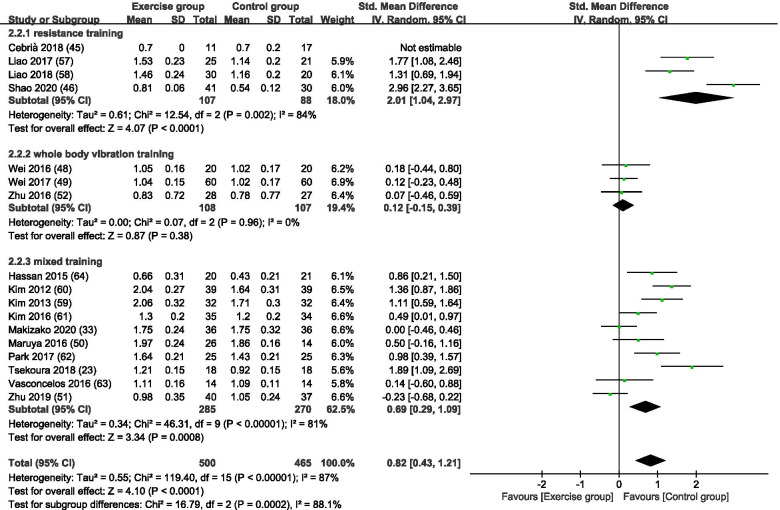
A forest plot of the subgroup analyses of 17 studies with GS as an outcome for sarcopenia based on different exercise training modes

Figure 5 is a forest plot of the subgroup analyses of 17 studies with GS as an outcome for sarcopenia based on different exercise training modes. Of the 17 studies in this subgroup, 4 studies applied RT [54, 55, 57, 64], 3 studies applied WBVT [51, 53, 61] and 10 studies applied MT. [16, 23, 33, 52, 63, 66–70] Collectively, 500 subjects were in exercise group and 465 were in the control group. With all exercise modes combined, the exercise group showed a significant increase in GS compared with the control group (SMD = 0.82, 95% CI: 0.43 to 1.21, *p* < 0.0001, I^2^ = 87%). In subgroup analysis, RT and MT showed a significant increase in GS compared with the control group (RT, SMD = 2.01, 95% CI: 1.04 to 2.97, *p* < 0.0001, I^2^ = 84%; MT, SMD = 0.69, 95% CI: 0.29 to 1.09, *p* = 0.0008, I^2^ = 81%). WBVT resulted in no significant difference in GS between the exercise and control groups (SMD = 0.12, 95% CI: − 0.15 to 0.39, *p* = 0.38, I^2^ = 0%).

**Fig. 6:**
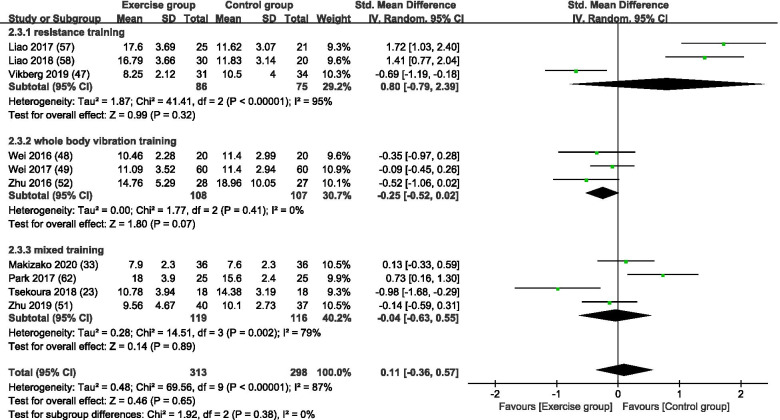
A forest plot of the subgroup analyses of 10 studies with CS as an outcome for sarcopenia based on different exercise training modes

Figure 6 is a forest plot of the subgroup analyses of 10 studies (3 RT [56, 57, 64], 3 WBVT [51, 53, 61], 4 MT [23, 33, 52, 68]) with CS as an outcome for sarcopenia based on different exercise training modes. Collectively, 313 subjects were in the exercise group and 298 were in the control group. With all exercise modes combined, there was no significant difference in CS times between the exercise and control group (SMD = 0.11, 95% CI: − 0.36 to 0.57, *p* = 0.65, I^2^ = 87%). Subgroup analysis showed no significant differences in CS times between the exercise and control groups for RT, WBVT, and MT (RT, SMD = 0.80, 95% CI: − 0.79 to 2.39, *p* = 0.32, I^2^ = 95%; WBVT, SMD = -0.25, 95% CI: − 0.52 to 0.02, *p* = 0.07, I^2^ = 0%; MT, SMD = -0.04, 95% CI: − 0.63 to 0.55, *p* = 0.89, I^2^ = 79%).

## Discussion

In this study, 26 studies (25 RCTs and one non-RCT) were examined using a systematic review and meta-analysis to compare the effects of RT, WBVT, and MT on muscle strength and physical performance in older people with sarcopenia. The results of our studies showed that RT can improve KES, TUG times and GS, but not CS times; WBVT have a positive effect on TUG times, but did not have a significant effect on KES, GS and CS times; The effect of MT (combinations of exercise modes such as resistance and balance training; resistance and endurance training; resistance, balance and gait training; resistance, balance and aerobic training; resistance and aerobic training) is similar with RT, which also can improve KES, TUG times and GS, but not CS times. As observed in this study, not all exercise modes improve all aspects of muscle strength and physical performance in older people with sarcopenia. These differences show the importance to identify exercise training modes that improve aspects of muscle strength and physical performance as needed most by older people with sarcopenia [79].

So far, RT is one of the most common mode of exercises used to prevent and/or delay the progression of sarcopenia in a variety of older populations. Previous studies showed that RT can improve KES [80–82], TUG times [83] and GS [84] in older people with sarcopenia, which is consistent with the result of our study. However, CS times was not been significantly improved by RT in our study. We speculated that different RT protocols of included RT studies may be a reason why RT has no significant effect on CS in our study. Given that several studies reviewed in this systematic review and meta-analysis included squats in RT studies [49, 56, 62], it is possible that the dose of exercise need to improve sit-to-stand performance was insufficient for older people with sarcopenia. And studies by Soligon et al. [85] and Vitale et al. [86] demonstrated that RT protocols including squat movements can significantly improve CS times, which may be due to the sufficient exercise dose and increased proficiency of squat movements of older people in our study. Thus, we speculated that RT protocols including squat movements is an important factor which may produce an effective influence on CS times.

Our systematic review and meta-analysis failed to show beneficial effects of WBVT on KES, GS and CS times, but it showed improvements on **TUG times** in older people with sarcopenia. The result of TUG times is consistent with previous studies. For instance, Chang et al. demonstrated that WBVT can improve TUG times in older people with sarcopenia [87]. But in addition to TUG times, the effects of WBVT on GS and CS times were without significant effects in our study. It indicated that TUG maybe more sensitive to exercise, and the change of TUG times is more significant than that of other physical performance indicators such as GS and CS times at the same time. However, previous studies were inconsistent with our study on the result of the effects of WBVT on GS and CS times. Wei et al. demonstrated that 12-week WBV exercise not only can make a positive improvement on TUG times, but also GS and CS times [51]. In terms of the intervention period, we found that the total vibration intervention period in our included WBVT studies averaged 10 weeks, and 12 weeks in Wei et al.’s study. Therefore, we speculated that the effect of WBVT on physical performance will be affected by time, and a suitable-long intervention period can comprehensively improve physical performance.

The fact of our meta-analysis failed to show a significant effect of WBVT on KES, but previous studies have opposite results for the effect of WBVT on KES in older people. For instance, a meta-analysis of 6 controlled clinical trials and RCTs by Wu et al. [88] and a meta-analysis of 12 studies (7 studies in younger people, mean ages of 11.8–37.7 yr.; and 5 studies with older people, mean ages 60.7–77.6 yr.) by Osawa et al. [89] both demonstrated that WBVT can lead to a significant improvement in lower body muscle strength as measured by KES in older people without and with sarcopenia [88, 89]. For such different results, it is speculated that different WBVT protocols may cause the differences of effects on KES. Firstly, the vibration frequency for the WBVT may have limited its ability to improve KES. In a study of eighty community-dwelling older adults with age-related muscle loss, Wei et al. compared different combinations of vibration frequencies (20 Hz, 40 Hz, 60 Hz) on knee extension performance. The results showed that the vibration frequency of 40 Hz was optimal in improving isokinetic knee extension performance [65]. However, in one third of the cited studies, the frequency of WBV was lower than 20 Hz, which is likely too low to improve muscle strength. Thus, the lower frequency of WBVT among the studies reviewed in this systematic review and meta-analysis is a possible reason for the lack of significance in showing WBVT effect on KES. Secondly, the amplitude of WBV may also be a reason why WBVT has a non-significant effect on KES. In a meta-analysis of WBVT on muscle strength, Marin et al. observed that studies using lower amplitudes (2–6 mm) showed less efficacy on muscle strength compared with studies using higher amplitudes (8–10 mm) [90]. There have five of the six studies reviewed used amplitudes less than 6 mm (3–5 mm), we speculated that the peak-to-peak displacement amplitude of the WBV was a factor for the lack of improvement on muscle strength. Thirdly, the exposure time of WBV can also be a reason why it cannot improve KES. A study by Da Silva-Grigoletto found that repeated 60-s bouts of WBVT with a total exposure time of 10 min was optimal than longer bout durations (i.e., 90 s) with a total exposure time greater than 10 min on improving muscle function [91]. Accordingly, it may be considered that muscle fatigue will occur when the exposure time is too excessive which could have limited the efficacy of WBVT on KES [92]. However, in the studies cited in our study, the exposure time of WBV varied from 60- to 180-s sets with a total exposure time of 5–15 min, and the different durations of WBV time ranging from 15 to 40 min. The above three point of views are why we speculate that WBVT cannot significantly improve KES, and more RCTs should be conducted to explore the effects of WBVT on muscle strength in older people with sarcopenia. Thus, clinical evidence will be added to sufficiently illustrate the effect of WBVT on sarcopenia.

The results of our meta-analysis showed that MT was effective on improving lower limb muscle strength as measured by KES, TUG times and GS in older people with sarcopenia, but not CS times. A systematic review and meta-analysis study showed improvements on TUG times from MT (combined with resistance and balance training) in community-dwelling frail older people [83], meanwhile several clinical studies also showed that the effect of MT improved significantly on TUG times [33, 93] which is consistent with the result of our study. However, there still have some studies showed that MT cannot make a significant improvement on TUG times. For instance, Wang et al. showed that MT program did not significantly improve TUG compared with usual care in aged 80 years or over older people with sarcopenia [32]. For the result of GS, it is speculated that the training movements is an extremely relevant factor, which makes MT have a significant impact on GS. In terms of training movements, MT have more abundant and complex movements. Pojskic et al. demonstrated that complex training protocols that require multi joint movements and whole body transitions over a short distance were effective in improving response time in agility-based activities of young trained athletes [94]. It is plausible subjects who performed complex motor skills during MT had faster reaction times and had the agility to navigate complex environmental conditions (e.g., stepping over objects, carrying items while walking) needed to increase GS following exercise training [95]. Complex exercise training increases vestibular-driven signals needed for postural changes and the ability to maintain balance during postural changes is an important ability for GS [96]. No significant changes in the CS times were observed following MT in older people with sarcopenia in our study. As the movement of CS reflects the comprehensive ability of lower limbs to raise and lower the body from a seated position [97], such movements may not always be integrated into exercise training protocols. An example of appropriate exercise movements in MT are the squat which requires strength of the quadriceps, gluteus maximus, and hamstrings muscles, and the bench press which builds core strength. Several studies reviewed in this systematic review and meta-analysis included squats in MT studies [16, 33, 49, 68] protocols showed that the dose of exercise to improve CS performance was insufficient for older people with sarcopenia.

In our study, RT and MT both have significant effects on KES, TUG times and GS, and this result is consistent with previous studies [32, 49, 72, 80, 81, 83, 98]. According to the forest plot of the subgroup analyses in RT and MT with KES, TUG and GS as outcomes, we found that RT maybe have a more effective influence than MT due to RT’s higher effect size than MT in each subgroup with KES, TUG, GS and CS. However, this is inconsistent with previous studies which showed that the effects of MT on muscle strength and physical performance were better than RT in healthy young man [73], or even MT have non-additive effect on muscle strength compared to the RT in athletes [74]. But so far, there have no studies exploring the comparison of RT and MT on muscle strength and physical performance in older people with sarcopenia. Thus, further research should conduct the comparison of these two modes exercises on muscle strength and physical performance in older people with sarcopenia.

In our study, WBVT only made a significant improvement on physical performance (TUG), but not effectively improved muscle strength. The above result we speculated that compared with MT, RT can improve muscle strength (KES) and physical performance (TUG times and GS) in older people with sarcopenia. Combined with above two speculations, the effect of WBVT on sarcopenia maybe not as good as RT and MT. To date, there have no studies to compare the effects of WBVT to RT or MT on sarcopenia, so according to the current findings, WBVT maybe not an effective alternative method for improving muscle strength and physical performance in older people with sarcopenia. This is mainly because there are few studies on the effect of WBVT on sarcopenia at present, so our study cannot include sufficient WBV studies that it is not enough to explain the effect of WBVT on sarcopenia. Therefore, further studies should be conducted to explore the effects of WBVT sarcopenia in older people.

### Limitations

This study had some limitations. First, we did not assess the effects of the different exercise training modes on muscle mass due to differences in criteria, indicators and assessment methods used to determine muscle mass. For example, studies using AWGS or EWGSOP criteria to measure skeletal muscle mass (SSM) or appendicular skeletal muscle mass (ASM) used different measurement methods to determine muscle mass (e.g., bioelectrical impedance analysis (BIA) or dual-energy X-ray absorptiometry [DXA]). These different measurement methods make it difficult to compare muscle mass between studies. Future studies in this field should unify the criteria, indicators and assessment methods used to measure muscle mass to allow between study comparisons. Second, the high heterogeneity in some results of this study (e.g., GS [I^2^ = 88%] and CS times [I^2^ = 88%]) might have been caused by differences in the assessment and exercise training programs. Researchers should conduct experiments in accordance with the standards and guidelines for assessing the studies independent and dependent variables to reduce the heterogeneity between studies and make them comparable. Third, we only included three exercise training modes (RT, MT, and WBVT) to explore the effects of different exercise training modes on muscle strength and physical performance outcomes in older people with sarcopenia. Other exercise training modes may prove beneficial in increasing muscle strength and physical performance. Moreover, differences in age, gender and/or factors related to aging may have moderated the effects of the three exercise training modes on the study outcomes in older people with sarcopenia. More studies should be conducted to explore the effects of additional exercise training modes and stratify the results by gender, age groups and gender-specific age groups to understand the effects of exercise training on muscle strength and physical performance in older people with sarcopenia.

## Conclusions

In older people with sarcopenia, the findings show that resistance training (RT) and mixed training (MT) exercise training modes have positive effects on knee extension strength (KES) and physical performance tests of the Timed Up and Go (TUG) and gait speed (GS), but did not improve performance in the Chair Stand (CS). Whole Body Vibration training (WBVT) had a positive effect on the TUG times, but had no effects on KES, GS and CS outcomes. Plausible reasons can explain these findings to include differences in the exercise training movements, exercise-specific demands on the body, and variations in exercise training protocols. Overall, RT, MT and WBVT are worthwhile exercise modes to achieve various improvements on muscle strength and physical performance in older adults with sarcopenia. RT alone or with MT combined with other exercise training modes such as aerobic, balance and gait training may be better than WBVT to improve overall physical function in older people, especially those who are frail. Older people with age-related disabilities (e.g., dementia, osteoarthritis, and hemiplegia) and who may have difficulty performing RT and MT exercise training modes may benefit from WBVT in standing or sitting postures to maintain and/or improve aspects of physical function. These findings should be confirmed by high-quality randomized controlled trials (RCTs) that explore the effects of different exercise trainings modes and protocols on muscle strength and physical performance.

## Supplementary Information

Additional file 1. (DOCX 24.6 kb)

## Data Availability

The datasets used and/or analysed during the current study are available from the corresponding author on reasonable request.

## Abbreviations

AWGS: Asian Working Group for Sarcopenia
EWGSOP: European Working Group on Sarcopenia in Older People
PEDro: Physiotherapy Evidence Database
PRISMA: Preferred Reporting Items for Systematic Review and Meta-Analysis
PROSPERO: International Prospective Register of Systematic Reviews site
GRADE: Grades of Recommendation, Assessment, Development and Evaluation
CNKI: China National Knowledge Infrastructure
NHANES III: Third National Health and Nutrition Examination Survey
CDC: Centers for Disease Control and Prevention
BIA: Bioelectrical impedance analysis
DXA: Dual energy X-ray absorptiometry
RT: Resistance training
VT: Vibration training
WBV: Whole body vibration training
WBVT: Whole body vibration training
MT: Mixed training
AT: Aerobic training
ET: Endurance training
ETG: Exercise training group
CG: Control group
RCT: Randomized controlled trial
RCTs: Randomized controlled trials
LG: Low frequency and long exercise time group
MG: Medium frequency and medium exercise time group
HG: High frequency and short exercise time group
ASM: Appendicular skeletal muscle mass
ASM/ht^2^: Appendicular skeletal muscle mass/height^2^
ALMI: Appendicular lean muscle mass index (ALMI = appendicular lean muscle mass/ height^2^ [ALM/m^2^])
AMI: Appendicular muscle mass index (AMI = appendicular muscle mass/height^2^ [AMM/m^2^])
AMM: Appendicular muscle mass
ASM*: = 0.2487(weight) + 0.0483(height)-0.1584(hip circumference) + 0.0732 (grip strength) + 2.5843(sex) + 5.8828
ASMI: Appendicular skeletal muscle mass index (ASMI = appendicular skeletal muscle mass/height^2^ [ASM/m^2^])
ASMI2: = appendicular skeletal muscle mass/weight^2^*100% [ASM/kg^2^]
ASMI4: = [0.244(weight) + 7.8(height) + 6.6(sex)-0.098(age) + race-3.3]/body mass index (ASM/BMI)
BMI: Body mass index
MM: Muscle mass
MMI: Muscle mass index (MMI = muscle mass/height^2^ [MMI/m^2^])
SMI: Skeletal muscle mass index (SMI = skeletal muscle mass/ height2 [SMI/m2])
SMM: Skeletal muscle mass
CS: Chair Stand
GS: Gait speed
HGS: Handgrip strength
KES: Knee extension strength
TUG: Timed Up and Go
4MWT: 4-m walk test
5-STS: 5-chair sit to stand test
6MWT: 6-m walk test
SPPB: Short Physical Performance Battery
ADL: Activities of daily living
UL: Upper limb
LL: Lower limb
M: Male
F: Female
W: Week
reps: Repetitions
RM: Repetition maximum
1RM: One repetition maximum
maxHR: Maximum Heart Rate
RPE: Rated perceived exertion
FNT: Maximum theoretical force
SD: Standard deviations
SMDs: Standardized mean differences
WMD: Weighted mean differences
95% CI: 95% confidence intervals
