# Supplementary material for: Effects of different exercise training modes on muscle strength and physical performance in older people with sarcopenia: a systematic review and meta-analysis
Source: BMC Geriatr. 2021 Dec 15;21:708. doi: 10.1186/s12877-021-02642-8 (PMC8672633; doi:10.1186/s12877-021-02642-8)
Supplement: Supplementary file 1 — Additional file 1. (DOCX 24.6 kb) [file 12877_2021_2642_MOESM1_ESM.docx]

**Pubmed:**

(((((((("Aging"[Mesh]) OR "Aged"[Mesh]) OR "Frailty"[Mesh]) OR "Frail Elderly"[Mesh]) OR ((((((((((((((((((((((((((((Aging[Title/Abstract]) OR (Senescence[Title/Abstract])) OR (Biological Aging[Title/Abstract])) OR (Aging, Biological[Title/Abstract])) OR (Aged[Title/Abstract])) OR (Elderly[Title/Abstract])) OR (Frailty[Title/Abstract])) OR (Frailties[Title/Abstract])) OR (geriatric[Title/Abstract])) OR (Frailness[Title/Abstract])) OR (Frailty Syndrome[Title/Abstract])) OR (Debility[Title/Abstract])) OR (Debilities[Title/Abstract])) OR (Frail Elderly[Title/Abstract])) OR (Elderly, Frail[Title/Abstract])) OR (Frail Elders[Title/Abstract])) OR (Elder, Frail[Title/Abstract])) OR (Elders, Frail[Title/Abstract])) OR (Frail Elder[Title/Abstract])) OR (Functionally-Impaired Elderly[Title/Abstract])) OR (Elderly, Functionally-Impaired[Title/Abstract])) OR (Functionally Impaired Elderly[Title/Abstract])) OR (Frail Older Adults[Title/Abstract])) OR (Adult, Frail Older[Title/Abstract])) OR (Adults, Frail Older[Title/Abstract])) OR (Frail Older Adult[Title/Abstract])) OR (Older Adult, Frail[Title/Abstract])) OR (Older Adults, Frail[Title/Abstract]))) AND (((("Sarcopenia"[Mesh]) OR "Muscle Weakness"[Mesh]) OR "Muscular Atrophy"[Mesh]) OR (((((((((((((((((((((((((((((((((((((((Sarcopenia[Title/Abstract]) OR (Sarcopenias[Title/Abstract])) OR (Sarcopenic[Title/Abstract])) OR (muscle loss[Title/Abstract])) OR (muscle wasting[Title/Abstract])) OR (Muscle Weakness[Title/Abstract])) OR (Muscle Weaknesses[Title/Abstract])) OR (Weakness, Muscle[Title/Abstract])) OR (Weaknesses, Muscle[Title/Abstract])) OR (Muscular Weakness[Title/Abstract])) OR (Muscular Weaknesses[Title/Abstract])) OR (Weakness, Muscular[Title/Abstract])) OR (Weaknesses, Muscular[Title/Abstract])) OR (muscular weakness[Title/Abstract])) OR (Muscular Atrophy[Title/Abstract])) OR (Atrophies, Muscular[Title/Abstract])) OR (Atrophy, Muscular[Title/Abstract])) OR (Muscular Atrophies[Title/Abstract])) OR (Atrophy, Muscle[Title/Abstract])) OR (Atrophies, Muscle[Title/Abstract])) OR (Muscle Atrophies[Title/Abstract])) OR (Muscle Atrophy[Title/Abstract])) OR (Neurogenic Muscular Atrophy[Title/Abstract])) OR (Atrophies, Neurogenic Muscular[Title/Abstract])) OR (Atrophy, Neurogenic Muscular[Title/Abstract])) OR (Muscular Atrophies, Neurogenic[Title/Abstract])) OR (Muscular Atrophy, Neurogenic[Title/Abstract])) OR (Neurogenic Muscular Atrophies[Title/Abstract])) OR (Neurotrophic Muscular Atrophy[Title/Abstract])) OR (Atrophies, Neurotrophic Muscular[Title/Abstract])) OR (Atrophy, Neurotrophic Muscular[Title/Abstract])) OR (Muscular Atrophies, Neurotrophic[Title/Abstract])) OR (Muscular Atrophy, Neurotrophic[Title/Abstract])) OR (Neurotrophic Muscular Atrophies[Title/Abstract])) OR (Age related muscle loss[Title/Abstract])) OR (Age-related muscle loss[Title/Abstract])) OR (muscle insufficiency[Title/Abstract])) OR (muscle depletion[Title/Abstract])) OR (skeletal muscle depletion[Title/Abstract])))) AND (((((((((((((((("Exercise"[Mesh]) OR "Motor Activity"[Mesh]) OR "Movement"[Mesh]) OR "Exercise Therapy"[Mesh]) OR "Motion Therapy, Continuous Passive"[Mesh]) OR "Exercise Movement Techniques"[Mesh]) OR "Physical Exertion"[Mesh]) OR "Exercise Tolerance"[Mesh]) OR "Endurance Training"[Mesh]) OR "Resistance Training"[Mesh]) OR "Weight Lifting"[Mesh]) OR "Circuit-Based Exercise"[Mesh]) OR "Vibration"[Mesh]) OR (((((((((((((((((((((((((((((((((((((((((((((((((((((((((((((((((((Exercise[Title/Abstract]) OR (Exercises[Title/Abstract])) OR (Physical Activity[Title/Abstract])) OR (Activities, Physical[Title/Abstract])) OR (Activity, Physical[Title/Abstract])) OR (Physical Activities[Title/Abstract])) OR (Exercise, Physical[Title/Abstract])) OR (Exercises, Physical[Title/Abstract])) OR (Physical Exercise[Title/Abstract])) OR (Physical Exercises[Title/Abstract])) OR (Acute Exercise[Title/Abstract])) OR (Acute Exercises[Title/Abstract])) OR (Exercise, Acute[Title/Abstract])) OR (Exercises, Acute[Title/Abstract])) OR (Exercise, Isometric[Title/Abstract])) OR (Exercises, Isometric[Title/Abstract])) OR (Isometric Exercises[Title/Abstract])) OR (Isometric Exercise[Title/Abstract])) OR (Exercise, Aerobic[Title/Abstract])) OR (Aerobic Exercise[Title/Abstract])) OR (Aerobic Exercises[Title/Abstract])) OR (Exercises, Aerobic[Title/Abstract])) OR (Exercise Training[Title/Abstract])) OR (Exercise Trainings[Title/Abstract])) OR (Training, Exercise[Title/Abstract])) OR (Trainings, Exercise[Title/Abstract])) OR (Activity[Title/Abstract])) OR (Activities[Title/Abstract])) OR (Motor Activity[Title/Abstract])) OR (Activities, Motor[Title/Abstract])) OR (Activity, Motor[Title/Abstract])) OR (Motor Activities[Title/Abstract])) OR (Sport[Title/Abstract])) OR (Sports[Title/Abstract])) OR (Movement[Title/Abstract])) OR (Movements[Title/Abstract])) OR (Exercise Therapy[Title/Abstract])) OR (Remedial Exercise[Title/Abstract])) OR (Exercise, Remedial[Title/Abstract])) OR (Exercises, Remedial[Title/Abstract])) OR (Remedial Exercises[Title/Abstract])) OR (Therapy, Exercise[Title/Abstract])) OR (Exercise Therapies[Title/Abstract])) OR (Therapies, Exercise[Title/Abstract])) OR (Rehabilitation Exercise[Title/Abstract])) OR (Exercise, Rehabilitation[Title/Abstract])) OR (Exercises, Rehabilitation[Title/Abstract])) OR (Rehabilitation Exercises[Title/Abstract])) OR (Motion Therapy[Title/Abstract])) OR (Motion Therapy, Continuous Passive[Title/Abstract])) OR (Movement Therapy, Continuous Passive[Title/Abstract])) OR (Passive Movement Therapy, Continuous[Title/Abstract])) OR (Continuous Passive Motion Therapy[Title/Abstract])) OR (Passive Motion Therapy, Continuous[Title/Abstract])) OR (Continuous Passive Movement Therapy[Title/Abstract])) OR (CPM Therapy[Title/Abstract])) OR (CPM Therapies[Title/Abstract])) OR (Exercise method[Title/Abstract])) OR (Pilates-Based Exercises[Title/Abstract])) OR (Pilates Based Exercises[Title/Abstract])) OR (Pilates Training[Title/Abstract])) OR (Kinesitherapy[Title/Abstract])) OR (exercise prescription[Title/Abstract])) OR (physical exertion[Title/Abstract])) OR (Exertion, Physical[Title/Abstract])) OR (Exertions, Physical[Title/Abstract])) OR (Efforts, Physical[Title/Abstract]))) OR (((((((((((((((((((((((((((((((((((((((aerobic training[Title/Abstract]) OR (cardio training[Title/Abstract])) OR (endurance exercise[Title/Abstract])) OR (Exercise Tolerance[Title/Abstract])) OR (Tolerance, Exercise[Title/Abstract])) OR (Endurance Training[Title/Abstract])) OR (Training, Endurance[Title/Abstract])) OR (Resistance Training[Title/Abstract])) OR (Training, Resistance[Title/Abstract])) OR (Strength Training[Title/Abstract])) OR (Training, Strength[Title/Abstract])) OR (Weight-Lifting Strengthening Program[Title/Abstract])) OR (Strengthening Program, Weight-Lifting[Title/Abstract])) OR (Strengthening Programs, Weight-Lifting[Title/Abstract])) OR (Weight Lifting Strengthening Program[Title/Abstract])) OR (Weight-Lifting Strengthening Programs[Title/Abstract])) OR (Weight-Lifting Exercise Program[Title/Abstract])) OR (Exercise Program, Weight-Lifting[Title/Abstract])) OR (Exercise Programs, Weight-Lifting[Title/Abstract])) OR (Weight Lifting Exercise Program[Title/Abstract])) OR (Weight-Lifting Exercise Programs[Title/Abstract])) OR (Weight-Bearing Strengthening Program[Title/Abstract])) OR (Strengthening Program, Weight-Bearing[Title/Abstract])) OR (Strengthening Programs, Weight-Bearing[Title/Abstract])) OR (Weight Bearing Strengthening Program[Title/Abstract])) OR (Weight-Bearing Strengthening Programs[Title/Abstract])) OR (Weight-Bearing Exercise Program[Title/Abstract])) OR (Exercise Program, Weight-Bearing[Title/Abstract])) OR (Exercise Programs, Weight-Bearing[Title/Abstract])) OR (Weight Bearing Exercise Program[Title/Abstract])) OR (strength training[Title/Abstract])) OR (strength exercise[Title/Abstract])) OR (Weight Lifting[Title/Abstract])) OR (Lifting, Weight[Title/Abstract])) OR (Balance training[Title/Abstract])) OR (mixed exercises[Title/Abstract])) OR (combined exercises[Title/Abstract])) OR (Multimodal exercise[Title/Abstract])) OR (Multimodal exercises[Title/Abstract]))) OR ((((((((((((((((((Vibration[Title/Abstract]) OR (Vibrations[Title/Abstract])) OR (vibration training[Title/Abstract])) OR (local muscle vibration[Title/Abstract])) OR (Whole body vibration[Title/Abstract])) OR (WBV[Title/Abstract])) OR (whole body vibration exercise[Title/Abstract])) OR (WBVE[Title/Abstract])) OR (Vibration therapy[Title/Abstract])) OR (vibrational therapy[Title/Abstract])) OR (vibrating therapy[Title/Abstract])) OR (vibration treatment[Title/Abstract])) OR (vibratory therapeutic[Title/Abstract])) OR (vibrotherapeutics[Title/Abstract])) OR (Vibrotactile[Title/Abstract])) OR (Vibration plate[Title/Abstract])) OR (vibrating platform[Title/Abstract])) OR (oscillating platforms[Title/Abstract])))) AND ((((((("Randomized Controlled Trials as Topic"[Mesh]) OR "Controlled Clinical Trials as Topic"[Mesh]) OR "Clinical Trials as Topic"[Mesh]) OR "Intention to Treat Analysis"[Mesh]) OR "Single-Blind Method"[Mesh]) OR "Double-Blind Method"[Mesh]) OR (((((((((((Randomized Controlled Trial[Publication Type]) OR (Controlled Clinical Trial[Publication Type])) OR (Clinical Trial[Publication Type])) OR ("random*"[Text Word])) OR (allocation[Text Word])) OR ("random allocation"[Text Word])) OR (placebo[Text Word])) OR (single blind[Text Word])) OR (double blind[Text Word])) OR ("randomized controlled trial*"[Text Word])) OR (RCT[Text Word])))) NOT (((((("Pulmonary Disease, Chronic Obstructive"[Mesh]) OR "Lung Diseases, Obstructive"[Mesh]) OR "Lung Diseases"[Mesh]) OR (((((((((((((((((((((((((((((Pulmonary Disease, Chronic Obstructive[Title/Abstract]) OR (Chronic Obstructive Lung Disease[Title/Abstract])) OR (Chronic Obstructive Pulmonary Diseases[Title/Abstract])) OR (COAD[Title/Abstract])) OR (COPD[Title/Abstract])) OR (Chronic Obstructive Airway Disease[Title/Abstract])) OR (Chronic Obstructive Pulmonary Disease[Title/Abstract])) OR (Airflow Obstruction, Chronic[Title/Abstract])) OR (Airflow Obstructions, Chronic[Title/Abstract])) OR (Chronic Airflow Obstructions[Title/Abstract])) OR (Chronic Airflow Obstruction[Title/Abstract])) OR (chronic obstructive lung disease[Title/Abstract])) OR (chronic lung disease[Title/Abstract])) OR (Lung Diseases, Obstructive[Title/Abstract])) OR (Lung Disease, Obstructive[Title/Abstract])) OR (Obstructive Lung Disease[Title/Abstract])) OR (Obstructive Lung Diseases[Title/Abstract])) OR (Obstructive Pulmonary Diseases[Title/Abstract])) OR (Obstructive Pulmonary Disease[Title/Abstract])) OR (Pulmonary Disease, Obstructive[Title/Abstract])) OR (Pulmonary Diseases, Obstructive[Title/Abstract])) OR (Lung Diseases[Title/Abstract])) OR (Disease, Lung[Title/Abstract])) OR (Diseases, Lung[Title/Abstract])) OR (Lung Disease[Title/Abstract])) OR (Pulmonary Disease[Title/Abstract])) OR (Disease, Pulmonary[Title/Abstract])) OR (Diseases, Pulmonary[Title/Abstract])) OR (Pulmonary Diseases[Title/Abstract]))) OR (("Neoplasms"[Mesh]) OR (((((((((((((((((((Neoplasms[Title/Abstract]) OR (Neoplasia[Title/Abstract])) OR (Neoplasias[Title/Abstract])) OR (Neoplasm[Title/Abstract])) OR (Tumors[Title/Abstract])) OR (Tumor[Title/Abstract])) OR (Cancer[Title/Abstract])) OR (Cancers[Title/Abstract])) OR (Malignancy[Title/Abstract])) OR (Malignancies[Title/Abstract])) OR (Malignant Neoplasms[Title/Abstract])) OR (Malignant Neoplasm[Title/Abstract])) OR (Neoplasm, Malignant[Title/Abstract])) OR (Neoplasms, Malignant[Title/Abstract])) OR (Benign Neoplasms[Title/Abstract])) OR (Neoplasms, Benign[Title/Abstract])) OR (Benign Neoplasm[Title/Abstract])) OR (Neoplasm, Benign[Title/Abstract])) OR (malignant neoplasm[Title/Abstract])))) OR (("Kidney Diseases"[Mesh]) OR (((((((Kidney Diseases[Title/Abstract]) OR (kidney disease[Title/Abstract])) OR (kidneys disease[Title/Abstract])) OR (kidneys diseases[Title/Abstract])) OR (Disease, Kidney[Title/Abstract])) OR (Diseases, Kidney[Title/Abstract])) OR (Kidney Disease[Title/Abstract]))))

**Cochrane Library:**

#1 MeSH descriptor: [Random Allocation] explode all trees

#2 MeSH descriptor: [Placebos] explode all trees

#3 MeSH descriptor: [Placebo Effect] explode all trees

#4 MeSH descriptor: [Randomized Controlled Trial] explode all trees

#5 MeSH descriptor: [Randomized Controlled Trials as Topic] explode all trees

#6 MeSH descriptor: [Controlled Clinical Trial] explode all trees

#7 MeSH descriptor: [Controlled Clinical Trials as Topic] explode all trees

#8 MeSH descriptor: [Clinical Trial] explode all trees

#9 MeSH descriptor: [Single-Blind Method] explode all trees

#10 MeSH descriptor: [Double-Blind Method] explode all trees

#11 (Randomized Controlled Trial OR Controlled Clinical Trial OR Clinical Trial):pt

#12 #1 OR #2 OR #3 OR #4 OR #5 OR #6 OR #7 OR #8 OR #9 OR #10 OR #11

#13 MeSH descriptor: [Aging] explode all trees

#14 MeSH descriptor: [Aged] explode all trees

#15 MeSH descriptor: [Geriatrics] explode all trees

#16 MeSH descriptor: [Frailty] explode all trees

#17 MeSH descriptor: [Frail Elderly] explode all trees

#18 (Aging OR Biological Aging OR Aging, Biological OR Senescence OR Aged OR Elderly OR Geriatric OR Geriatrics OR Gerontology OR Frailty OR Debility OR Debilities OR Frailty Syndrome OR Frailties OR Frailness OR Frail Elderly OR Elderly, Functionally-Impaired OR Functionally-Impaired Elderly OR Functionally Impaired Elderly OR Adults, Frail Older OR Older Adult, Frail OR Frail Older Adults OR Frail Older Adult OR Adult, Frail Older OR Older Adults, Frail OR Frail Elders OR Elders, Frail OR Frail Elder OR Elderly, Frail OR Elder, Frail):ti,ab,kw

#19 #13 OR #14 OR #15 OR #16 OR #17 OR #18

#20 MeSH descriptor: [Sarcopenia] explode all trees

#21 MeSH descriptor: [Muscle Weakness] explode all trees

#22 MeSH descriptor: [Muscular Atrophy] explode all trees

#23 (Sarcopenia OR Sarcopenias OR Sarcopenic OR Presarcopenia OR muscle loss OR muscle wasting OR Muscle Weakness OR Weakness, Muscular OR Muscular Weaknesses OR Weaknesses, Muscular OR Weaknesses, Muscle OR Muscle Weaknesses OR Muscular Weakness OR Weakness, Muscle OR muscular weakness OR Muscular Atrophy OR Neurogenic Muscular Atrophy OR Atrophy, Neurogenic Muscular OR Atrophies, Neurotrophic Muscular OR Neurogenic Muscular Atrophies OR Neurotrophic Muscular Atrophy OR Muscular Atrophies, Neurotrophic OR Neurotrophic Muscular Atrophies OR Muscular Atrophy, Neurogenic OR Atrophies, Neurogenic Muscular OR Atrophy, Neurotrophic Muscular OR Muscular Atrophies, Neurogenic OR Muscular Atrophy, Neurotrophic OR Atrophies, Muscle OR Muscle Atrophy OR Muscle Atrophies OR Atrophy, Muscular OR Atrophies, Muscular OR Muscular Atrophies OR Atrophy, Muscle OR Age related muscle loss OR Age-related muscle loss OR muscle insufficiency OR muscle depletion):ti,ab,kw

#24 #20 OR #21 OR #22 OR #23

#25 MeSH descriptor: [Exercise] explode all trees

#26 MeSH descriptor: [Motor Activity] explode all trees

#27 MeSH descriptor: [Sports] explode all trees

#28 MeSH descriptor: [Movement] explode all trees

#29 MeSH descriptor: [Exercise Therapy] explode all trees

#30 MeSH descriptor: [Motion Therapy, Continuous Passive] explode all trees

#31 MeSH descriptor: [Exercise Movement Techniques] explode all trees

#32 MeSH descriptor: [Endurance Training] explode all trees

#33 MeSH descriptor: [Resistance Training] explode all trees

#34 MeSH descriptor: [Weight Lifting] explode all trees

#35 MeSH descriptor: [Compulsive Exercise] explode all trees

#36 MeSH descriptor: [Exercise Tolerance] explode all trees

#37 MeSH descriptor: [Vibration] explode all trees

#38 (Exercise OR Exercises, Isometric OR Exercise, Isometric OR Isometric Exercises OR Isometric Exercise OR Acute Exercise OR Acute Exercises OR Exercises, Acute OR Exercise, Acute OR Exercise, Aerobic OR Aerobic Exercise OR Exercises, Aerobic OR Aerobic Exercises OR Activities, Physical OR Physical Exercises OR Exercises OR Physical Activities OR Activity, Physical OR Exercises, Physical OR Physical Exercise OR Physical Activity OR Exercise, Physical OR Trainings, Exercise OR Exercise Trainings OR Training, Exercise OR Exercise Training OR Exercises OR Activity OR Motor Activity OR Activity, Motor OR Activities, Motor OR Motor Activities OR Activities OR Sports OR Sport OR Athletic OR Athletics OR Movement OR Movements OR Exercise Therapy OR Remedial Exercises OR Remedial Exercise OR Exercise Therapies OR Therapies, Exercise OR Rehabilitation Exercises OR Exercises, Remedial OR Exercises, Rehabilitation OR Exercise, Rehabilitation OR Rehabilitation Exercise OR Exercise, Remedial OR Therapy, Exercise OR Motion Therapy OR Motion Therapy, Continuous Passive OR Passive Motion Therapy, Continuous OR Continuous Passive Motion Therapy OR Exercise method OR Exercise Movement Techniques OR Exercises, Pilates-Based OR Training, Pilates OR Pilates-Based Exercises OR Pilates Based Exercises OR Pilates Training OR Movement Techniques, Exercise OR Exercise Movement Technics OR movement techniques OR Therapeutic Exercise OR Kinesitherapy OR exercise prescription OR physical exertion OR aerobic training OR cardio training OR Endurance Training OR Training, Endurance OR endurance exercise OR Resistance Training OR Weight-Lifting Strengthening Program OR Weight-Lifting Exercise Program OR Weight-Lifting Strengthening Programs OR Strengthening Program, Weight-Lifting OR Weight Lifting Strengthening Program OR Weight Lifting Exercise Program OR Exercise Programs, Weight-Lifting OR Weight-Lifting Exercise Programs OR Exercise Program, Weight-Lifting OR Strengthening Programs, Weight-Lifting OR Training, Strength OR Strength Training OR Training, Resistance OR Exercise Programs, Weight-Bearing OR Strengthening Programs, Weight-Bearing OR Weight-Bearing Strengthening Programs OR Weight Bearing Strengthening Program OR Exercise Program, Weight-Bearing OR Weight-Bearing Strengthening Program OR Weight-Bearing Exercise Programs OR Strengthening Program, Weight-Bearing OR Weight-Bearing Exercise Program OR Weight Bearing Exercise Program OR progressive resistance OR strength training OR strength exercise OR weight training OR Weight Lifting OR Liftings, Weight OR Lifting, Weight OR Weight Liftings OR Balance training OR mixed exercises OR Compulsive Exercise OR Exercise, Compulsive OR Exercise Tolerance OR Tolerance, Exercise OR combined exercises OR Multimodal exercise OR Multimodal exercises OR Vibration OR Vibrations OR vibration training OR local muscle vibration OR Whole body vibration OR WBV OR whole body vibration exercise OR WBVE OR Vibration therapy OR vibrational therapy OR vibrating therapy OR vibration treatment OR vibratory therapeutic OR vibrotherapeutics OR Vibrotactile OR Vibration plate OR vibrating platform OR oscillating platforms):ti,ab,kw

#39 #25 OR #26 OR #27 OR #28 OR #29 OR #30 OR #31 OR #32 OR #33 OR #34 OR #35 OR #36 or #37 OR #38

#40 MeSH descriptor: [Pulmonary Disease, Chronic Obstructive] explode all trees

#41 MeSH descriptor: [Lung Diseases] explode all trees

#42 (COPD OR Pulmonary Disease, Chronic Obstructive OR Chronic Airflow Obstructions OR Airflow Obstruction, Chronic OR Airflow Obstructions, Chronic OR Chronic Airflow Obstruction OR COAD OR Chronic Obstructive Pulmonary Disease OR Chronic Obstructive Airway Disease OR Chronic Obstructive Pulmonary Diseases OR COPD OR Chronic Obstructive Lung Disease OR chronic obstructive lung disease OR Lung Diseases OR Pulmonary Diseases OR Disease, Lung OR Diseases, Lung OR Pulmonary Disease OR Disease, Pulmonary OR Diseases, Pulmonary OR Lung Disease OR chronic lung disease OR Pulmonary Disease, Chronic Obstructive):ti,ab,kw

#43 #40 OR #41 OR #42

#44 MeSH descriptor: [Neoplasms] explode all trees

#45 (Cancer OR Neoplasms OR Neoplasm, Malignant OR Malignancies OR Cancer OR Malignancy OR Malignant Neoplasms OR Malignant Neoplasm OR Cancers OR Neoplasms, Malignant OR Neoplasias OR Tumors OR Tumor OR Neoplasia OR Neoplasm OR Benign Neoplasms OR Neoplasms, Benign OR Benign Neoplasm OR Neoplasm, Benign OR malignant neoplasm):ti,ab,kw

#46 #44 OR #45

#47 MeSH descriptor: [Kidney Diseases] explode all trees

#48 (kidney disease OR kidneys disease OR Kidney Diseases OR Disease, Kidney OR Diseases, Kidney OR Kidney Disease):ti,ab,kw

#49 #47 OR #48

#50 #43 OR #46 OR #49

#51 #12 AND #19 AND #24 AND #39 NOT #50

**Embase:**

#23. #3 AND #6 AND #9 AND #12 NOT #22

#22. #15 OR #18 OR #21

#21. #19 OR #20

#20. 'kidneys diseases':ab,ti OR 'kidneys disease':ab,ti OR 'kidney disease':ab,ti OR 'kidney diseases':ab,ti

#19. 'kidneys diseases'/exp

#18. #16 OR #17

#17. 'malignant neoplasm':ab,ti OR 'cancer':ab,ti OR 'cancers':ab,ti OR 'malignant neoplasia':ab,ti OR 'malignant neoplastic disease':ab,ti OR 'malignant tumor':ab,ti OR 'malignant tumour':ab,ti OR 'neoplasia, malignant':ab,ti OR 'tumor, malignant':ab,ti OR 'tumour, malignant':ab,ti

#16. 'malignant neoplasm'/exp

#15. #13 OR #14

#14. 'chronic obstructive lung disease':ab,ti OR 'chronic airflow obstruction':ab,ti OR 'chronic airway obstruction':ab,ti OR 'chronic obstructive bronchitis':ab,ti OR 'chronic obstructive bronchopulmonary disease':ab,ti OR 'chronic obstructive lung disorder':ab,ti OR 'chronic obstructive pulmonary disease':ab,ti OR 'chronic obstructive pulmonary disorder':ab,ti OR 'chronic obstructive respiratory disease':ab,ti OR 'copd':ab,ti OR 'lung chronic obstructive disease':ab,ti OR 'lung disease, chronic obstructive':ab,ti OR 'lung diseases, obstructive':ab,ti OR 'obstructive lung disease':ab,ti OR 'obstructive lung disease, chronic':ab,ti OR 'obstructive lung diseases':ab,ti OR 'obstructive pulmonary disease':ab,ti OR 'obstructive respiratory disease':ab,ti OR 'obstructive respiratory tract disease':ab,ti OR 'pulmonary disease, chronic obstructive':ab,ti OR 'pulmonary disorder, chronic obstructive':ab,ti OR 'chronic lung disease':ab,ti OR 'chronic pneumopathy':ab,ti OR 'chronic pulmonary disease':ab,ti OR 'lung disease, chronic':ab,ti

#13. 'chronic obstructive lung disease'/exp OR 'chronic lung disease'/exp

#12. #10 OR #11

#11. 'biometric exercise':ab,ti OR 'effort':ab,ti OR 'exercise capacity':ab,ti OR 'exercise performance':ab,ti OR 'exertion':ab,ti OR 'fitness training':ab,ti OR 'physical conditioning, human':ab,ti OR 'physical effort':ab,ti OR 'exercises':ab,ti OR 'activity':ab,ti OR 'motor activity':ab,ti OR 'activity, motor':ab,ti OR 'activities':ab,ti OR 'competitive gymnastics':ab,ti OR 'competitive sport':ab,ti OR 'sports':ab,ti OR 'sport':ab,ti OR 'physical exercise':ab,ti OR 'physical exercises':ab,ti OR 'physical activity':ab,ti OR 'activity, physical':ab,ti OR 'physical activities':ab,ti OR 'exercise training':ab,ti OR 'exercise trainings':ab,ti OR 'movement':ab,ti OR 'movement therapy':ab,ti OR 'continuous passive motion therapy':ab,ti OR 'motion therapy':ab,ti OR 'motion therapy, continuous passive':ab,ti OR 'exercise method':ab,ti OR 'movement techniques':ab,ti OR 'kinesiotherapy':ab,ti OR 'corrective exercise':ab,ti OR 'exercise movement techniques':ab,ti OR 'exercise therapy':ab,ti OR 'exercise treatment':ab,ti OR 'kinesiotherapeutic intervention':ab,ti OR 'kinesiotherapeutic method':ab,ti OR 'kinesiotherapeutic procedure':ab,ti OR 'kinesiotherapeutic technique':ab,ti OR 'kinesiotherapeutical treatment':ab,ti OR 'kinesitherapeutic exercises':ab,ti OR 'kinesitherapeutic intervention':ab,ti OR 'kinesitherapeutic method':ab,ti OR 'kinesitherapeutic methodology':ab,ti OR 'kinesitherapeutic procedure':ab,ti OR 'kinesitherapeutic technique':ab,ti OR 'kinesitherapeutic treatment':ab,ti OR 'kinesitherapeutical treatment':ab,ti OR 'kinesitherapy':ab,ti OR 'sktm (specialized kinesitherapeutic methodology)':ab,ti OR 'specialised kinesitherapeutic methodology':ab,ti OR 'specialized kinesitherapeutic methodology':ab,ti OR 'therapeutic exercise':ab,ti OR 'therapy, exercise':ab,ti OR 'treatment, exercise':ab,ti OR 'exercise prescription':ab,ti OR 'physical exertion':ab,ti OR 'exercise':ab,ti OR 'acute exercise':ab,ti OR 'acute exercises':ab,ti OR 'isometric exercise':ab,ti OR 'exercise, isometric':ab,ti OR 'isometric endurance':ab,ti OR 'isometric endurance test':ab,ti OR 'isometric training':ab,ti OR 'isometric exercises':ab,ti OR 'aerobic exercise':ab,ti OR 'aerobic dance':ab,ti OR 'aerobic dancing':ab,ti OR 'aerobics':ab,ti OR 'aerobics exercise':ab,ti OR 'dancing, aerobic':ab,ti OR 'exercise, aerobic':ab,ti OR 'low impact aerobic exercise':ab,ti OR 'low impact aerobics':ab,ti OR 'step aerobics':ab,ti OR 'aerobic exercises':ab,ti OR 'aerobic training':ab,ti OR 'cardio training':ab,ti OR 'endurance training':ab,ti OR 'endurance exercise':ab,ti OR 'endurance exercise training':ab,ti OR 'resistance exercise':ab,ti OR 'resistance exercise training':ab,ti OR 'weight bearing exercise':ab,ti OR 'progressive resistance':ab,ti OR 'strength training':ab,ti OR 'resistance training':ab,ti OR 'strength exercise':ab,ti OR 'weight training':ab,ti OR 'weight lifting':ab,ti OR 'lifting, weight':ab,ti OR 'balance training':ab,ti OR 'balance training system':ab,ti OR 'balance training device':ab,ti OR 'smart balance master':ab,ti OR 'mixed exercises':ab,ti OR 'combined exercises':ab,ti OR 'multimodal exercise':ab,ti OR 'multimodal exercises':ab,ti OR 'vibration response':ab,ti OR 'vibrations':ab,ti OR 'vibration training':ab,ti OR 'wbv exercise':ab,ti OR 'wbv therapy':ab,ti OR 'wbv training':ab,ti OR 'wbve (whole body vibration exercise)':ab,ti OR 'wbvt (whole body vibration training)':ab,ti OR 'whole body vibrating training':ab,ti OR 'whole body vibration (therapy)':ab,ti OR 'whole body vibration exercise':ab,ti OR 'whole body vibration exercises':ab,ti OR 'whole body vibration therapy':ab,ti OR 'whole body vibrational therapy':ab,ti OR 'wholebody vibration (therapy)':ab,ti OR 'wholebody vibration exercise':ab,ti OR 'wholebody vibration training':ab,ti OR 'local muscle vibration':ab,ti OR 'vibration':ab,ti OR 'whole body vibration （wbv）':ab,ti OR 'whole body vibrations':ab,ti OR 'wholebody vibration':ab,ti OR 'whole body vibration exercise (wbve)':ab,ti OR 'whole body vibration training':ab,ti OR 'electrovibratory massage':ab,ti OR 'microvibration therapy':ab,ti OR 'vibrating massage':ab,ti OR 'vibration massage':ab,ti OR 'vibrational massage':ab,ti OR 'vibrator massage':ab,ti OR 'vibrator therapy':ab,ti OR 'vibratory massage':ab,ti OR 'vibratory therapy':ab,ti OR 'vibro-massage':ab,ti OR 'vibroacustic massage':ab,ti OR 'vibromassage':ab,ti OR 'vibrational therapy':ab,ti OR 'vibrating therapy':ab,ti OR 'vibration treatment':ab,ti OR 'vibratory therapeutic':ab,ti OR 'vibrotherapeutics':ab,ti OR 'vibrotherapy':ab,ti OR 'vibration therapy':ab,ti OR 'vibration plate':ab,ti OR 'vibrating platform':ab,ti OR 'oscillating platforms':ab,ti

#10. 'exercise'/exp OR 'activity'/exp OR 'motor activity'/exp OR 'sport'/exp OR 'physical activity'/exp OR 'movement therapy'/exp OR 'kinesiotherapy'/exp OR 'acute exercise'/exp OR 'isometric exercise'/exp OR 'aerobic exercise'/exp OR 'aerobic training'/exp OR 'endurance training'/exp OR 'resistance training'/exp OR 'strength exercise'/exp OR 'weight training'/exp OR 'weight lifting'/exp OR 'balance training'/exp OR 'vibration'/exp OR 'vibrations'/exp OR 'whole body vibration training'/exp OR 'whole body vibration'/exp OR 'vibration therapy'/exp

#9. #7 OR #8

#8. sarcopenia:ab,ti OR sarcopenias:ab,ti OR sarcopenic:ab,ti OR presarcopenia:ab,ti OR 'muscle loss':ab,ti OR 'muscle weakness':ab,ti OR 'muscle strength loss':ab,ti OR 'muscle weakening':ab,ti OR 'muscular insufficiency':ab,ti OR 'neuromuscular fatigue':ab,ti OR 'weakness, muscle':ab,ti OR 'muscular weakness':ab,ti OR 'amyotrophia':ab,ti OR 'amyotrophy':ab,ti OR 'atrophic muscular disorders':ab,ti OR 'atrophy type 2':ab,ti OR 'atrophy, muscle':ab,ti OR 'degeneration, muscle':ab,ti OR 'hirayama disease':ab,ti OR 'muscle atrophia':ab,ti OR 'muscle cell degeneration':ab,ti OR 'muscle degeneration':ab,ti OR 'muscle fiber atrophy':ab,ti OR 'muscle fiber degeneration':ab,ti OR 'muscle recession':ab,ti OR 'muscle wasting':ab,ti OR 'muscular atrophy':ab,ti OR 'muscular degeneration':ab,ti OR 'muscular disorders, atrophic':ab,ti OR 'myoatrophy':ab,ti OR 'myodegeneration':ab,ti OR 'myofibrillar degeneration':ab,ti OR 'myophagism':ab,ti OR 'age related muscle loss':ab,ti OR 'age-related muscle loss':ab,ti OR 'muscle insufficiency':ab,ti OR 'muscle depletion':ab,ti

#7. 'sarcopenia'/exp OR 'muscle loss'/exp OR 'muscle atrophy'/exp OR 'muscle weakness'/exp

#6. #4 OR #5

#5. 'aging':ab,ti OR 'age induced changes':ab,ti OR 'ageing':ab,ti OR 'aging biology':ab,ti OR 'aging process':ab,ti OR 'aging rate':ab,ti OR 'aged':ab,ti OR 'aged patient':ab,ti OR 'aged people':ab,ti OR 'aged person':ab,ti OR 'aged subject':ab,ti OR 'elderly':ab,ti OR 'elderly patient':ab,ti OR 'elderly people':ab,ti OR 'elderly person':ab,ti OR 'elderly subject':ab,ti OR 'senior citizen':ab,ti OR 'senium':ab,ti OR 'geriatric':ab,ti OR 'frailty':ab,ti OR 'frail elderly':ab,ti

#4. 'aging'/exp OR 'aged'/exp OR 'geriatric'/exp OR 'frailty'/exp OR 'frail elderly'/exp

#3. #1 OR #2

#2. random*:ab,ti OR allocation:ab,ti OR placebo:ab,ti OR rct:ab,ti OR 'randomised controlled trial*':ab,ti OR 'randomized controlled trial*':ab,ti OR 'clinical trial*':ab,ti OR 'single blind':ab,ti OR 'double blind':ab,ti

#1. 'randomization'/exp OR 'placebo'/exp OR 'placebo effect'/exp OR 'randomized controlled trial'/exp OR 'randomized controlled trial (topic)'/exp OR 'controlled clinical trial'/exp OR 'clinical trial'/exp OR 'clinical trial (topic)'/exp OR 'single blind procedure'/exp OR 'double blind procedure'/exp

**Web of Science:**

#1 TS=(random OR allocation OR random allocation OR placebo OR single blind OR single blind method OR double blind OR double blind method OR randomized controlled trial OR randomised controlled trial OR RCT OR clinical trial)

#2 TS=(Aging OR Senescence OR Biological Aging OR Aging, Biological OR Aged OR Elderly OR Frailty OR Frailties OR geriatric OR Frailness OR Frailty Syndrome OR Debility OR Debilitie OR Frail Elderly OR Elderly, Frail OR Frail Elders OR Elder, Frail OR Elders, Frail OR Frail Elder OR Functionally-Impaired Elderly OR Elderly, Functionally-Impaired OR Functionally Impaired Elderly OR Frail Older Adults OR Adult, Frail Older OR Adults, Frail Older OR Frail OR Older Adult OR Older Adult, Frail OR Older Adults, Frail)

#3 TS=(Sarcopenia OR Sarcopenias OR Sarcopenic OR muscle loss OR muscle wasting OR Muscle Weakness OR Muscle Weaknesses—Weakness, Muscle OR Weaknesses, Muscle OR Muscular Weakness OR Muscular Weaknesses OR Weakness, Muscular OR Weaknesses, Muscular OR muscular weakness OR Muscular Atrophy OR Atrophies, Muscular OR Atrophy, Muscular OR Muscular Atrophies OR Atrophy, Muscle OR Atrophies, Muscle OR Muscle Atrophies OR Muscle Atrophy OR Neurogenic Muscular Atrophy OR Atrophies, Neurogenic Muscular OR Atrophy, Neurogenic Muscular OR Muscular Atrophies, Neurogenic OR Muscular Atrophy, Neurogenic OR Neurogenic Muscular Atrophies OR Neurotrophic Muscular Atrophy OR Atrophies, Neurotrophic Muscular OR Atrophy, Neurotrophic Muscular OR Muscular Atrophies, Neurotrophic OR Muscular Atrophy, Neurotrophic OR Neurotrophic Muscular Atrophies OR Age related muscle loss OR Age-related muscle loss OR muscle insufficiency OR muscle depletion OR skeletal muscle depletion)

#4 TS=(Exercise OR Exercises OR Physical Activity OR Activities, Physical OR Activity, Physical OR Physical Activities OR Exercise, Physical OR Exercises, Physical OR Physical Exercise OR Physical Exercises OR Acute Exercise OR Acute Exercises OR Exercise, Acute OR Exercises, Acute OR Exercise, Isometric OR Exercises, Isometric OR Isometric Exercises OR Isometric Exercise OR Exercise, Aerobic OR Aerobic Exercise OR Aerobic Exercises OR Exercises, Aerobic OR Exercise Training OR Exercise Trainings OR Training, Exercise OR Trainings, Exercise OR Activity OR Activities OR Motor Activity OR Activities, Motor OR Activity, Motor OR Motor Activities OR Sport OR Sports OR Movement OR Movements OR Exercise Therapy OR Remedial Exercise OR Exercise, OR Remedial OR Exercises, Remedial OR Remedial Exercises OR Therapy, Exercise OR Exercise Therapies OR Therapies, Exercise OR Rehabilitation Exercise OR Exercise, Rehabilitation OR Exercises, OR Rehabilitation OR Rehabilitation Exercises OR Motion Therapy OR Motion Therapy, Continuous Passive OR Movement Therapy, Continuous Passive OR Passive Movement Therapy, Continuous OR Continuous Passive Motion Therapy OR Passive Motion Therapy, Continuous OR Continuous Passive Movement Therapy OR CPM Therapy OR CPM Therapies OR Therapies, CPM OR Therapy, CPM OR Exercise method OR Exercise Movement Techniques OR Movement Techniques, Exercise OR Exercise Movement Technics OR Pilates-Based Exercises OR Exercises, Pilates-Based OR Pilates Based Exercises OR Pilates Training OR Training, Pilates OR movement techniques OR Therapeutic Exercise OR Kinesitherapy OR exercise prescription OR physical exertion OR Exertion, Physical OR Exertions, Physical OR Physical Exertions OR Physical Effort OR Effort, Physical OR Efforts, Physical OR Physical Efforts OR aerobic training OR cardio training OR endurance exercise OR Exercise Tolerance OR Tolerance, Exercise OR Endurance Training OR Training, Endurance OR Resistance Training OR Training, Resistance OR Strength Training OR Training, Strength OR Weight-Lifting Strengthening Program OR Strengthening Program, Weight-Lifting OR Strengthening Programs, Weight-Lifting OR Weight Lifting Strengthening Program OR Weight-Lifting Strengthening Programs OR Weight-Lifting Exercise Program OR Exercise Program, Weight-Lifting OR Exercise Programs, Weight-Lifting OR Weight Lifting Exercise Program OR Weight-Lifting Exercise Programs OR Weight-Bearing Strengthening Program OR Strengthening Program, Weight-Bearing OR Strengthening Programs, Weight-Bearing OR Weight Bearing Strengthening Program OR Weight-Bearing Strengthening Programs OR Weight-Bearing Exercise Program OR Exercise Program, Weight-Bearing OR Exercise Programs, Weight-Bearing OR Weight Bearing Exercise Program OR Weight-Bearing Exercise Programs OR strength training OR strength exercise OR weight training OR Weight Lifting OR Lifting, Weight OR Liftings, Weight OR Weight Liftings OR Balance training OR Circuit-Based Exercise OR Circuit Based Exercise OR Circuit-Based Exercises OR Exercise, Circuit-Based OR Exercises, Circuit-Based OR Circuit Training OR Training, Circuit OR mixed exercises OR combined exercises OR Multimodal exercise OR Multimodal exercises OR Vibration OR Vibrations OR vibration training OR local muscle vibration OR Whole body vibration OR WBV OR whole body vibration exercise OR WBVE OR Vibration therapy OR vibrational therapy OR vibrating therapy OR vibration treatment OR vibratory therapeutic OR vibrotherapeutics OR Vibrotactile OR Vibration plate OR vibrating platform OR oscillating platforms)

#5 TS=(Pulmonary Disease, Chronic Obstructive OR Chronic Obstructive Lung Disease OR Chronic Obstructive Pulmonary Diseases OR COAD OR COPD OR Chronic Obstructive Airway Disease OR Chronic Obstructive Pulmonary Disease OR Airflow Obstruction, Chronic OR Airflow Obstructions, Chronic OR Chronic Airflow Obstructions OR Chronic Airflow Obstruction OR chronic obstructive lung disease OR chronic lung disease OR Lung Diseases, Obstructive OR Lung Disease, Obstructive OR Obstructive Lung Disease OR Obstructive Lung Diseases OR Obstructive Pulmonary Diseases OR Obstructive Pulmonary Disease OR Pulmonary Disease, Obstructive OR Pulmonary Diseases, Obstructive OR Lung Diseases OR Disease, Lung OR Diseases, Lung OR Lung Disease OR Pulmonary Disease OR Disease, Pulmonary OR Diseases, Pulmonary OR Pulmonary Diseases)

#6 TS=(Neoplasms OR Neoplasia OR Neoplasias OR Neoplasm OR Tumors OR Tumor OR Cancer OR Cancers OR Malignancy OR Malignancies OR Malignant Neoplasms OR Malignant Neoplasm OR Neoplasm, Malignant OR Neoplasms, Malignant OR Benign Neoplasms OR Neoplasms, Benign OR Benign Neoplasm OR Neoplasm, Benign OR malignant neoplasm)

#7 TS=(kidneys disease OR kidneys diseases OR Kidney Diseases OR Disease, Kidney OR Diseases, Kidney OR Kidney Disease)

#8 #7 OR #6 OR #5

#9 #1 AND #2 AND #3 AND #4 NOT #8
